# The aliphatic amidase AmiE is involved in regulation of *Pseudomonas aeruginosa* virulence

**DOI:** 10.1038/srep41178

**Published:** 2017-01-24

**Authors:** Thomas Clamens, Thibaut Rosay, Alexandre Crépin, Teddy Grandjean, Takfarinas Kentache, Julie Hardouin, Perrine Bortolotti, Anke Neidig, Marlies Mooij, Mélanie Hillion, Julien Vieillard, Pascal Cosette, Joerg Overhage, Fergal O’Gara, Emeline Bouffartigues, Alain Dufour, Sylvie Chevalier, Benoit Guery, Pierre Cornelis, Marc G. J. Feuilloley, Olivier Lesouhaitier

**Affiliations:** 1 Laboratory of Microbiology Signals and Microenvironment LMSM EA 4312, Normandie Univ, UNIROUEN, Evreux, France; 2 Univ. Bretagne-Sud, EA 3884, LBCM, IUEM, Lorient, France; 3 Univ. Lille, CHU Lille, EA 7366 - Recherche Translationnelle: relations hôte pathogènes, Lille, France; 4 Laboratory « Polymères, Biopolymères, Surfaces » (UMR 6270 CNRS), Proteomic Platform PISSARO, Normandie Univ, UNIROUEN, Mont-Saint-Aignan, France; 5 Karlsruhe Institute of Technology (KIT), Institute of Functional Interfaces, PO Box 3640, Karlsruhe, Germany; 6 BIOMERIT Research Centre, University College Cork, Cork, Ireland; 7 Normandie Univ, UNIROUEN, INSA Rouen, CNRS, COBRA (UMR 6014), Evreux, France; 8 School of Biomedical Sciences, Curtin Health Innovation Research Institute, Curtin University, Perth, Australia

## Abstract

We have previously shown that the eukaryotic C-type natriuretic peptide hormone (CNP) regulates *Pseudomonas aeruginosa* virulence and biofilm formation after binding on the AmiC sensor, triggering the *amiE* transcription. Herein, the involvement of the aliphatic amidase AmiE in *P. aeruginosa* virulence regulation has been investigated. The proteome analysis of an AmiE over-producing strain (AmiE^+^) revealed an expression change for 138 proteins, including some that are involved in motility, synthesis of quorum sensing compounds and virulence regulation. We observed that the AmiE^+^ strain produced less biofilm compared to the wild type, and over-produced rhamnolipids. In the same line, AmiE is involved in *P. aeruginosa* motilities (swarming and twitching) and production of the quorum sensing molecules *N*-acyl homoserine lactones and Pseudomonas Quinolone Signal (PQS). We observed that AmiE overproduction reduced levels of HCN and pyocyanin causing a decreased virulence in different hosts (i.e. *Dictyostelium discoideum* and *Caenorhabditis elegans*). This phenotype was further confirmed in a mouse model of acute lung infection, in which AmiE overproduction resulted in an almost fully virulence decrease. Taken together, our data suggest that, in addition to its role in bacterial secondary metabolism, AmiE is involved in *P. aeruginosa* virulence regulation by modulating pilus synthesis and cell-to-cell communication.

Bacteria in general and opportunistic pathogens in particular must adapt their physiology to the microenvironment in which they live. *Pseudomonas aeruginosa* is an opportunistic pathogen that is a leading cause of mortality for cystic fibrosis patients^1^,^2^. This highly adaptable pathogen can perceive host environment modifications and respond rapidly which implies that it is able to detect a large range of signals including eukaryotic ones^3^,^4^. Detection of compounds that are present in the bacterial micro-environment is often mediated by a sensor protein associated to a signal transduction pathway of varying degrees of complexity allowing an appropriate answer to environmental changes^5^,^6^. The detection of external environment changes by these sensor systems enables bacteria to react and adapt their metabolism. *P. aeruginosa*, a metabolically versatile organism, possesses a large number of genes encoding sensor proteins or associated proteins involved in signal transduction in its genome^7^. Next to the sensing of nutrients for uptake and consumption of carbon sources^8^, bacteria can sense eukaryotic signals released in their micro-environment^9^. This is particularly relevant for human pathogens that need to adapt their metabolism, stress resistance and virulence factors production in the host tissues to enhance the infectious process. Even if the large genome of *P. aeruginosa* encodes numerous sensor proteins^7^, the vast number of compounds encountered during an infectious process suggests that other bacterial proteins, besides their well-known primary function, could have alternative functions either in the detection of chemical compounds or in the regulation of the response^10^. Indeed, AmiC which was first identified for its ability to bind acetamide^11^ has recently been shown to be a sensor for the human C-type natriuretic peptide (CNP) hormone with a pharmacological profile that was similar to its human orthologue^12^.

The *amiC* gene lies within an operon composed of five genes in *P. aeruginosa* PAO1 (*amiEBCRS*) and of four genes in *P. aeruginosa* PA14 (*amiEBCR*)^13^. The function of the *ami* operon products is to allow hydrolysis of short-chain aliphatic amides to their corresponding organic acids by the aliphatic amidase AmiE. This enzymatic activity is triggered by acetamide that is used as carbon and nitrogen sources by *P. aeruginosa*. This action is regulated by AmiC and AmiR which are both neutralized in the absence of signal^11^,^13^. The binding of acetamide to AmiC leads to the release of AmiR, which acts as a transcriptional anti-termination factor allowing the transcription of the full *ami* operon. Therefore, although it is devoid of phosphorylation capacity, AmiR is nevertheless considered to act as the response regulator of the *ami* system^14^. In addition, the translation of the *amiE* mRNA is controlled by the carbon catabolite repression proteins Hfq and Crc^15^. Recently, it has been shown that the human hormone CNP acts as an AmiC agonist, which activates the transcription of the whole *ami* operon, including the gene for the AmiE aliphatic enzyme^12^. Since CNP is able to modify *P. aeruginosa* virulence^16^ and biofilm formation^12^, we hypothesized that the final product of the *ami* operon, i.e. the AmiE enzyme, could be involved in balancing biofilm formation and bacterial virulence.

The goal of the present study was therefore to investigate these AmiE potential alternative functions in *P. aeruginosa.* To carry out this study we decided to construct a strain that overproduces AmiE and compare it to the wild type strain, rather than comparing an *amiE* mutant to the wild type strain since the AmiE protein level in the latter might be low as a consequence of the *amiE* translation repression by the Crc and Hfq proteins^15^,^17^,^18^. The proteomic analysis of a strain over-producing AmiE indicates an involvement of AmiE in *P. aeruginosa* motility, production of quorum sensing (QS) signal molecules, virulence regulation and biofilm formation. These observations were validated by phenotypic characterizations showing that over-production of AmiE altered biofilm formation, swarming capacity, and production of both *N*-acyl homoserine lactones (AHLs) and the Pseudomonas Quinolone Signal (PQS), which are the QS signal molecules. Using several models, we demonstrated that AmiE over-production resulted in decreased *P. aeruginosa* virulence both *in vitro* and *in vivo*, suggesting that AmiE is not only involved in carbon-nitrogen metabolic processes.

## Results

### Proteome profile of *P. aeruginosa* PA14 AmiE^+^ strain versus PA14 control strain

**Figure 1:**
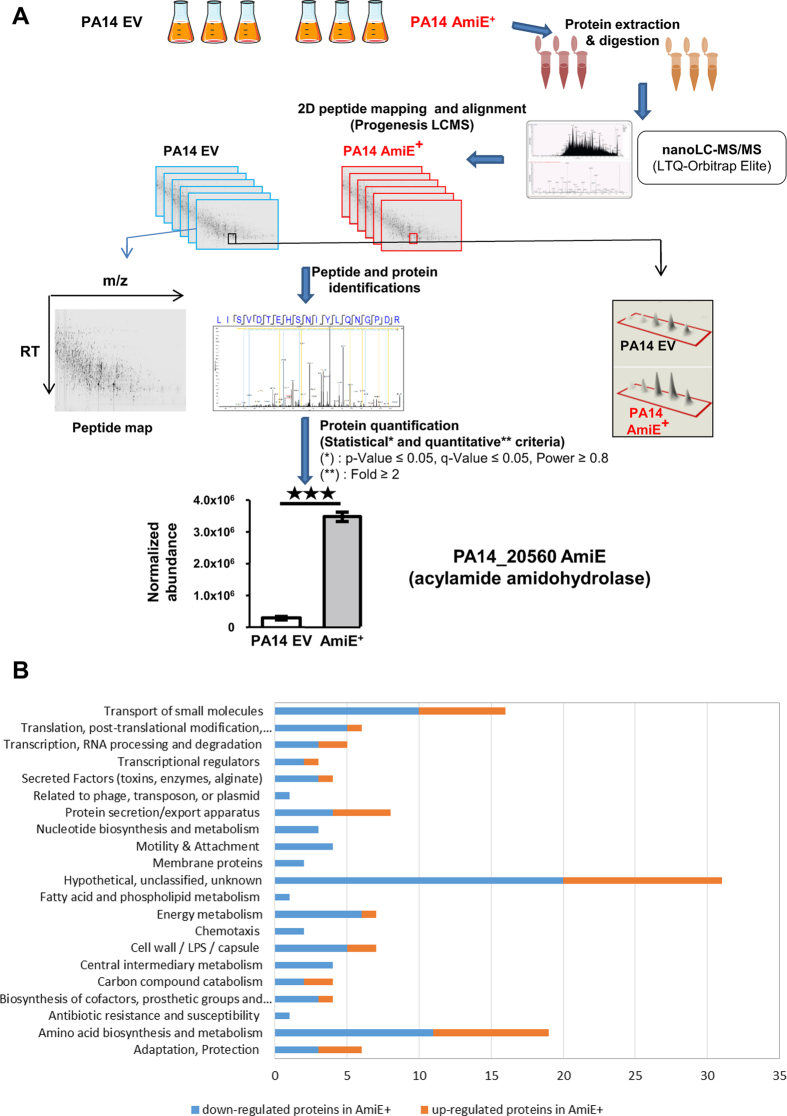
Whole bacterial proteome analysis. (**A**) Workflow explaining the strategy used for quantification of the whole proteome of both PA14 EV and AmiE^+^ strains using nanoLC-MS/MS (LTQ-Orbitrap Elite). (**B**) Functional classes of AmiE^+^ -regulated protein expression. All the 138 proteins that had a significant difference in production between PA14 EV and AmiE^+^ strains were classified according to their function. Functional classes were determined using functional classifications manually assigned by PseudoCAP (www.pseudomonas.com; ref. 27).

We investigated the impact of AmiE over-production on the whole *P. aeruginosa* PA14 proteome. To this aim, proteomes from PA14 wild type (PA14 WT), the strain over-producing AmiE (AmiE^+^) that is PA14 WT carrying the *amiE* gene inserted into the pBBR-MCS5 plasmid, and the strain harboring the empty pBBR-MCS5 vector (PA14 EV) were compared using ultrahigh-resolution liquid chromatography-tandem mass-spectrometry (nLC-ESI-MS/MS) on a hybrid linear ion trap LTQ Orbitrap instrument (Fig. 1A). Since no difference was observed between the PA14 WT strain and the PA14 EV strain (data not shown), we therefore subsequently compared the AmiE^+^ strain to the PA14 EV strain only. Peptide analyses revealed that 138 proteins were differentially produced between PA14 EV and AmiE^+^ with at least a two-fold change (Supplementary Table S1). Among them, 43 were over-produced in AmiE^+^ strain including AmiE itself by a factor 11.8 (Fig. 1A), which confirms that adding the *amiE* gene on a multicopy plasmid indeed led to AmiE protein over-production. On the opposite, 95 proteins were down-regulated in AmiE^+^ compared to PA14 EV (Supplementary Table S1).

Among the proteins differentially produced between the PA14 EV and AmiE^+^ strains, we observed proteins involved in the transport of small molecules, transcriptional regulators or secreted factors, proteins known to be involved in secretion/export apparatus, proteins of the cell wall/LPS/capsule and other proteins involved in adaptation/protection (Fig. 1B and Supplementary Table S1). In parallel, we noticed that several proteins that were less present in AmiE^+^ are involved in bacterial motility (Fig. 1B). This prompted us to assay motility as well as other phenotypes related to virulence and biofilm formation.

### Involvement of AmiE in *P. aeruginosa* motility

**Figure 2:**
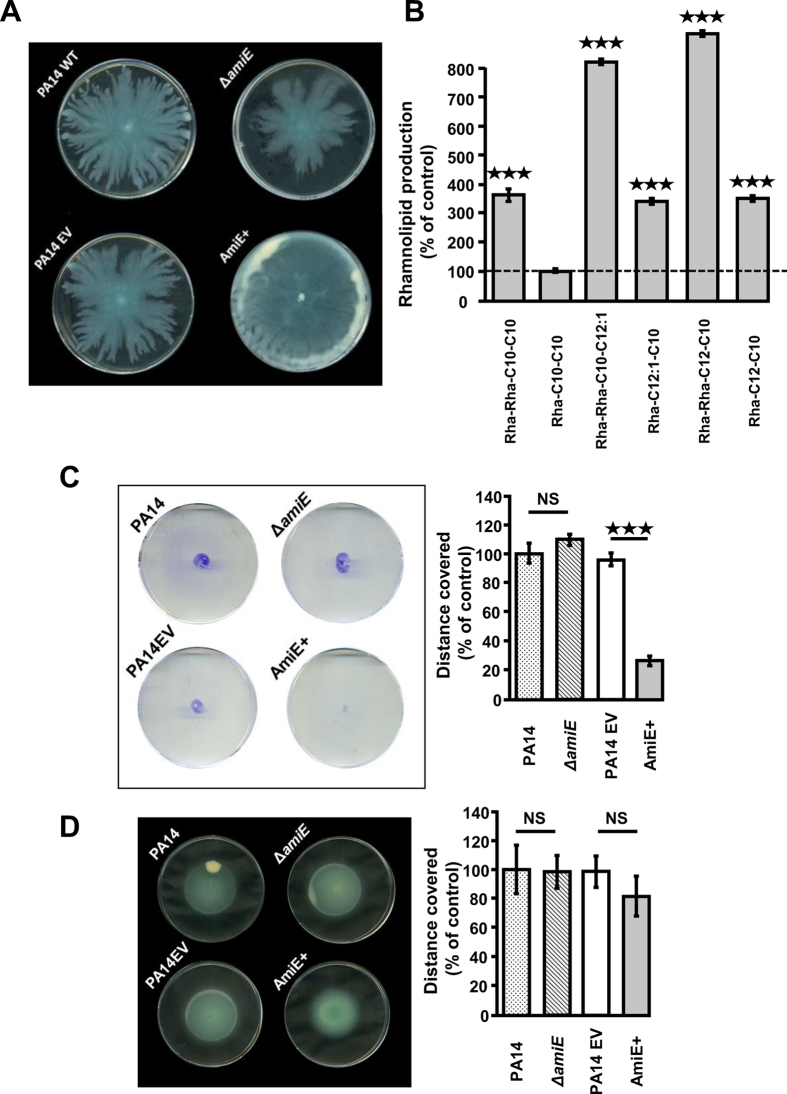
AmiE involvement in *P. aeruginosa* motility. (**A**) Pictures show swarming motilities of *P. aeruginosa* PA14 WT (control), PA14 Δ*amiE* mutant strain, PA14 strain harboring the empty vector (PA14 EV), and PA14 AmiE over-producing strain (AmiE^+^) after 18 h at 37 °C. (**B**) Amounts of rhamnolipids produced by *P. aeruginosa* AmiE^+^ relative to the production by the control *P. aeruginosa* PA14 EV (dotted line). Data are the mean of three independent experiments. (**C**) Pictures show twitching motilities of *P. aeruginosa* strains after 16 h at 37 °C. Graphs show diameter of colonies spread by twitching motility in comparison with the wild type strain. (**D**) Pictures show swimming motilities of *P. aeruginosa* strains after 16 h at 37 °C. Graphs show diameter of colonies spread by swimming motility in comparison with the wild type strain. All results for motilities are representative of four independent experiments each performed in triplicate. ^★★★^*P* < 0.001; NS: Not significantly different.

Since some proteins involved in motility were found in lower amounts in the AmiE^+^ strain, we investigated the motility phenotypes of the different strains. *P. aeruginosa* displays three types of motilities, i.e. swimming that involves the activity of the flagellum, swarming that requires flagella, type IV pili and the rhamnolipids biosurfactants, and twitching that is mainly related to type IV pilus activity. We observed that the AmiE^+^ strain presented a hyper-swarmer phenotype compared to the PA14 EV strain, whereas the Δ*amiE* mutant displayed a slightly decreased ability to swarm (Fig. 2A). Accordingly, the production of the major rhamnolipid species (Rha-Rha-C10-C10, Rha-Rha-C10-C12:1, Rha-C12:1-C10, Rha-Rha-C12-C10 and Rha-C12-C10) was strongly enhanced (i.e. by 3.6, 8.1, 3.4, 9.1 and 3.5 time, respectively) in AmiE^+^ compared to the PA14EV strain (Fig. 2B), with only the amount of the Rha-C10-C10 rhamnolipid being unchanged, suggesting that the hyper-swarmer phenotype of AmiE^+^ was linked to the over-production of rhamnolipids (Fig. 2B). Accordingly, we observed that the rhamnosyltransferase chain A enzyme RhlA, which is required for rhamnolipid biosynthesis was over-produced (6.5 fold) in AmiE^+^ compared to PA14 EV (Supplementary Figure S1B). By contrast, the twitching motility was strongly impaired in AmiE^+^ compared to the PA14 WT, PA14 EV and Δ*amiE* strains (Fig. 2C). This phenotype was in line with the decreased amounts of the type 4 fimbrial biogenesis proteins PilY1, PilQ and the type IV pilin structural subunit, which were reduced by about 551, 2.6 and 40 fold, respectively (Supplementary Figure S1). In line with this, the AmiE^+^ strain was totally resistant to the PO_4_ phages that require functional type IV pili for infecting *P. aeruginosa* (Supplementary Figure S3). No difference in swimming ability was observed between all the investigated strains (PA14 WT, PA14 EV, Δ*amiE* and AmiE^+^) (Fig. 2D). Altogether, these data suggest that the over-production of AmiE resulted in altered motilities such as enhanced swarming, which could at least partly be explained by an increased production of rhamnolipids, and lack of twitching that can be related to decreased production of type IV pili.

### Involvement of AmiE in biofilm formation

**Figure 3:**
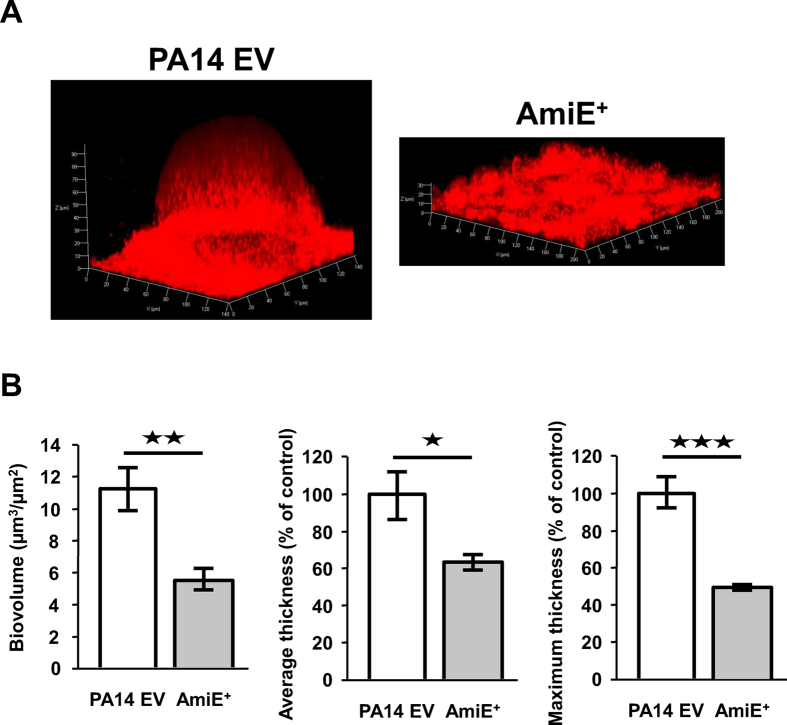
AmiE and *P. aeruginosa* biofilm formation. (**A**) 3D shadow representations of the biofilms developed by *P. aeruginosa* PA14 EV and AmiE^+^ under dynamic conditions at 37 °C for 24 h in LB broth. Biofilms were stained with Syto 61 red dye and observed by confocal laser scanning microscopy. (**B**) COMSTAT analyses of biofilms of *P. aeruginosa* PA14 EV and AmiE^+^ strains. Data are the mean of eighteen measures from six independent channels from two independent experiments. ^★★★^*P* < 0.001; ^★★^*P* < 0.01; NS: Not significantly different.

As swarming ability and biofilm formation are inversely correlated^19^,^20^, we decided to investigate the involvement of AmiE in *P. aeruginosa* biofilm formation. We checked the biofilm formation capacity on glass slides under a dynamic flow, as previously described^12^. Under these conditions, the AmiE^+^ strain formed a biofilm that was lacking the typical mushroom-like structures that were displayed by the PA14 EV biofilms (Fig. 3A). A COMSTAT analysis showed that maximal and average thicknesses of AmiE^+^ biofilms were respectively reduced by 51.6 ± 1.8% and 36.5 ± 4.2% compared to the values obtained for the PA14 EV biofilms (Fig. 3B). Finally, the biovolume, reflecting the biofilm biomass, was reduced by about two-fold in the case of AmiE^+^ compared to PA14 EV (5.7 ± 0.7 μm^3^/μm^2^ and 11.3 ± 1.4 μm^3^/μm^2^, respectively) (Fig. 3B). In parallel, we evaluated the ability of the strains to form microcolonies in liquid artificial sputum medium (ASM). We observed that the AmiE^+^ strain was impaired in the formation of tight microcolonies at the bottom of the wells compared to the wild type (Supplementary Figure S4), suggesting a possible alteration of the self-aggregation phenotype in ASM medium.

### Production of QS molecules

**Figure 4:**
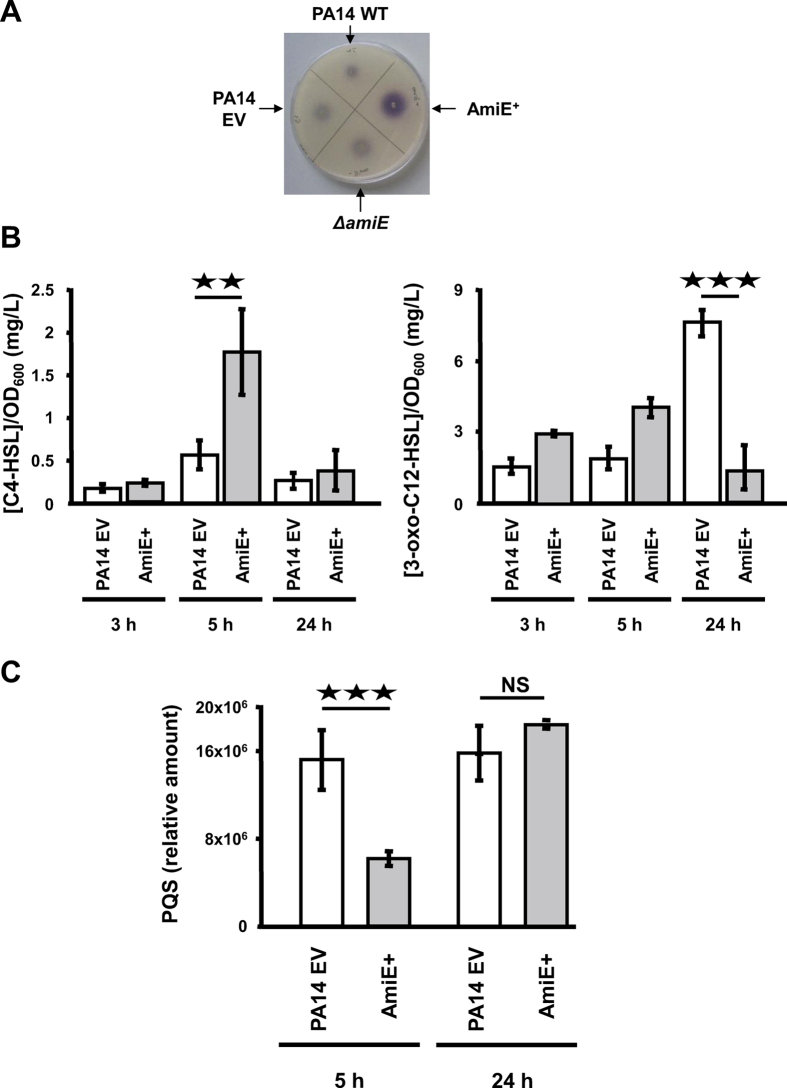
AmiE involvement in production of QS molecules. (**A**) AHL production by the indicated *P. aeruginosa* strains. *C. violaceum* CV026 was used as a biosensor for AHL detection. An overnight culture of *C. violaceum* CV026 was mixed in a ratio 1:70 (v/v) with LB medium agar and each *P. aeruginosa* strain was spotted on the agar medium and incubated during 24 h at 37 °C. Three independent experiments have been realized resulting in the same pattern. (**B**) Comparison of C_4_-HSL (left) and of 3-oxo-C_12_-HSL (right) production by *P. aeruginosa* AmiE^+^ and PA14 EV at 3 h, 5 h and 24 h of growth. (**C**) Comparison of PQS production by *P. aeruginosa* AmiE^+^ and PA14 EV at 5 h and 24 h of growth. Data are the mean of three independent experiments. ^★★★^*P* < 0.001; ^★★^*P* < 0.01; NS: Not significantly different.

Knowing that *P. aeruginosa* swarming motility and hence rhamnolipid production are controlled by *N*-acyl-homoserine lactones (AHLs)^21^,^22^, and in line with the increase of the two AHL synthases LasI and RhlI in the AmiE^+^ strain (Supplementary Figure S1 and Supplementary Table S1), we decided to investigate the effect of AmiE over-production in QS signal production. To this aim, the colorimetric assay based on the AHL biosensor *Chromobacterium violaceum* CVO26, which mainly detects short-chains AHLs, was first performed on LB agar plates. As shown in Fig. 4A, the AmiE^+^ colonies were surrounded by a strong purple halo after 24 h of growth, suggesting that this strain over-produced *N*-butyryl-l-homoserine lactone (C_4_-HSL)^23^ synthesized by RhlI, whereas the three other strains PA14 WT, PA14 EV and Δ*amiE* produced lower, but similar amounts of C_4_-HSL (Fig. 4A). To get further insights into this phenotype, AHLs were extracted at different growth stages and analyzed by liquid chromatography-mass spectrometry (LCMS) as previously described^24^. In agreement with the biosensor results, the AmiE^+^ strain synthesized higher amounts of C_4_-HSL compared to the PA14 EV strain, which was especially visible after 5 h of growth (1.8 ± 0.5 mg.l^−1^.OD_600_^−1^ versus 0.6 ± 0.2 mg.l^−1^.OD_600_^−1^) (Fig. 4B and Supplementary Figure S2). Noticeably, the C_4_-HSL biosynthesis enzyme RhlI was found to be 2.3 time more abundant in this strain (Supplementary Table S1 and Supplementary Figure S1). This result is also consistent with the large over-production of rhamnolipids in the AmiE^+^ strain (Fig. 2B) and the higher amount of RhlA in AmiE^+^ (Supplementary Figure S1B), since C_4_-HSL and the corresponding Rhl QS system directly control the expression of genes responsible for rhamnolipid synthesis such as *rhlA*^25^. As for the production of other QS molecules, the AmiE^+^ strain produced about twice as much *N*-(3-oxododecanoyl)-l-homoserine lactone 3-oxo-C_12_-HSL compared to the PA14 EV strain (4.0 ± 0.4 mg.l^−1^.OD_600_^−1^ versus 1.9 ± 0.5 mg.l^−1^.OD_600_^−1^) after 5 h of growth (Fig. 4B), a phenotype that is consistent with the large over-production (by about 8 fold) of the biosynthetic enzyme LasI (Supplementary Table S1 and Supplementary Figure S1C). In striking contrast, the 3-oxo-C_12_-HSL amount was further decreased in supernatants of 24 h AmiE^+^ cultures, whereas it was increased in 24 h PA14 EV cultures (1.5 ± 0.9 mg.l^−1^.OD_600_^−1^ versus 7.6 ± 0.5 mg.l^−1^.OD_600_^−1^) (Fig. 4B). Finally, the quantification of PQS, the third QS signal, revealed that AmiE over-production impaired PQS synthesis during the late exponential growth phase of bacteria (5 h) while this difference was levelled down when *P. aeruginosa* reached the late stationary phase (24 h) (Fig. 4C). Accordingly, our proteomic analysis showed that after 5 h of growth the levels of PQS biosynthetic enzymes, PqsC, PqsB and PqsD were lower in the AmiE^+^ strain compared to PA14 EV (Supplementary Figure S1A). Taken together, our data showed that over-production of AmiE results in deep alterations in QS signals levels, especially by increasing C4-HSL production and decreasing 3-oxo-C_12_-HSL, altering the balance between the two AHL signal molecules.

### Involvement of AmiE in virulence

**Figure 5:**
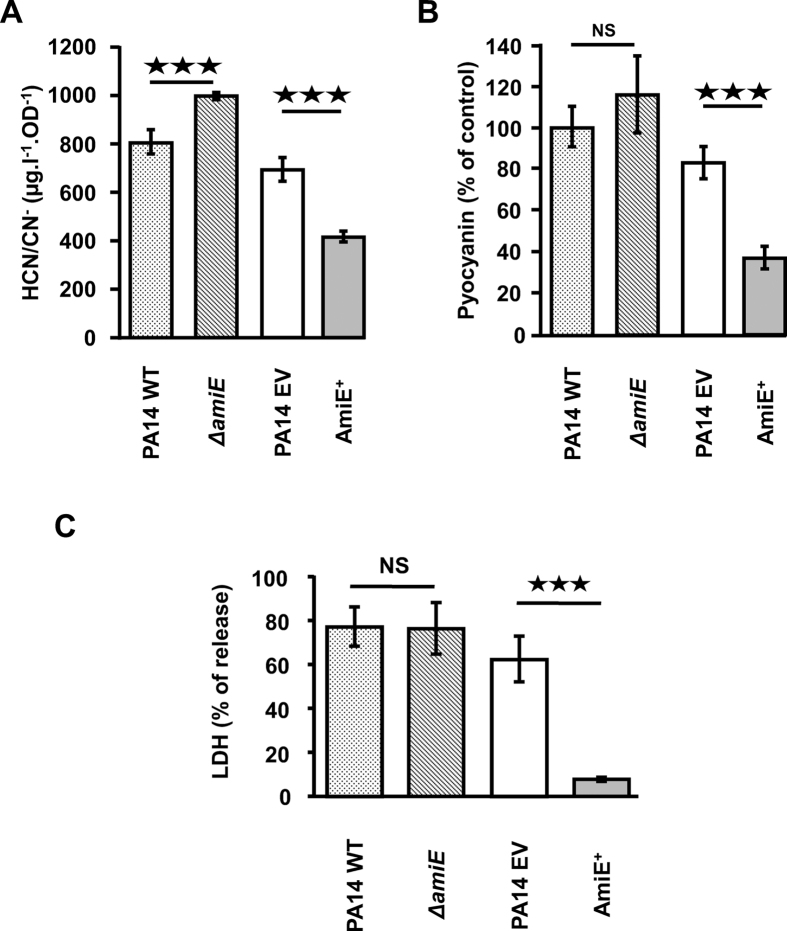
AmiE involvement in toxin production and cytotoxic activity. (**A**) Relative amounts of HCN/CN^−^ in supernatants of *P. aeruginosa* strains culture. The mean HCN/CN^−^ level in the control was 1,834 ± 134 μg.l^−1^. (**B**) Relative amounts of pyocyanin in supernatants of *P. aeruginosa* strains culture. (**C**) Involvement of AmiE in *P. aeruginosa* cytotoxicity towards A549 lung cells. The measurement of LDH was done after 6 h of contact with PA14 WT, Δ*amiE*, PA14 EV and AmiE^+^ strains. All data are the mean of three independent experiments. ^★★★^*P* < 0.001.

Since QS regulates *P. aeruginosa* virulence through modulation of virulence factors production^26^, we next checked the production of two major virulence factors: HCN and the phenazine pigment pyocyanin. In line with the reduced amount of the HcnC protein in the AmiE^+^ strain (Supplementary Figure S1A), the production of HCN by AmiE^+^ was found to be decreased compared to PA14 EV (−39%; *P* < 0.001) (Fig. 5A). Inversely, the production of HCN in the Δ*amiE* strain was slightly (+24%) but significantly (*P* < 0.001) enhanced (Fig. 5A). Pyocyanin production was also significantly reduced in AmiE^+^ compared to PA14 WT and PA14 EV (−61% and −53%, respectively; *P* < 0.001) (Fig. 5B), in agreement with the decreased accumulation of the pyocyanin biosynthetic enzymes PhzB1 and PhzE (Supplementary Table S1), and of the QS major signal PQS that is involved in pyocyanin production^27^ (Supplementary Table S1 and Fig. 4C). We therefore examined the cytotoxicity of AmiE^+^ on human A549 lung cells. In line with the above data, the PA14 AmiE^+^ strain displayed a very strong and highly significant decrease in cytotoxicity (*P* < 0.0001) (Fig. 5C). By contrast, the cytotoxicity of the Δ*amiE* mutant strain was similar to the control strain PA14 wild type (Fig. 5C).

**Figure 6:**
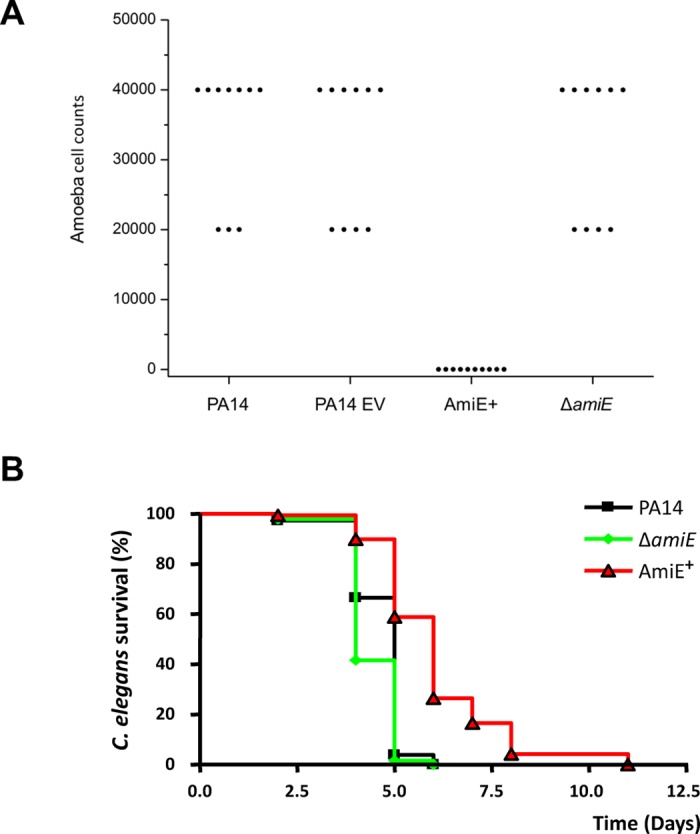
AmiE involvement in *P. aeruginosa* virulence. (**A**) *Dictyostelium discoideum* plate killing assay. Each point represents the number of amoebae required to form a plaque on the bacterial lawn of *P. aeruginosa* PA14 strains after 5 days of incubation. The AmiE^+^ strain has a total defect in this virulence model of infection, which was statistically significant as measured with the Mann Whitney test (^★★★^*P* < 0.001), n = 10), when compared with the PA14 EV strain or the Δ*amiE* strain. Data represent 10 samples from three independent experiments. (**B**) Kaplan-Meier survival plots of worms in contact with *P. aeruginosa* PA14 WT (n = 146) (black squares), AmiE^+^ (n = 138) (red triangle) or Δ*amiE* (n = 132) (green diamonds). Each value reported for the assay is the mean of measurements of nine samples from three independent experiments. Pairwise comparisons (log rank test) by strain: PA14 WT versus AmiE^+^, *P* < 0.0001; PA14 WT versus Δ*amiE, P* = 0.7914.

In a next step, *in vivo* virulence was assayed on two alternative eukaryotic infection models: the amoeba *Dictyostelium discoideum* (Fig. 6A) and the nematode worm *Caenorhabditis elegans* (Fig. 6B). In both cases, the over-production of AmiE resulted in a strongly decreased virulence of the AmiE^+^ strain, compared to PA14 WT, PA14 EV and PA14 Δ*amiE*. More precisely, the PA14 WT, PA14 EV and Δ*amiE* strains displayed a similar virulence level against *D. discoideum*, since a high number of amoebae were required to observe bacterial clearance (Fig. 6A). Conversely, the AmiE^+^ virulence was strongly and significantly reduced (*P* < 0.001) by more than 1,000-fold (Fig. 6A). The *C. elegans* nematodes are particularly sensitive to both HCN and pyocyanin^28^. Accordingly, growing *C. elegans* on PA14 WT lawns led to the death of about 95% of the worms after five days (Fig. 6B). Over-production of AmiE slowed down the lethality since almost 60% of worms were still alive after five days (*P* < 0.0001) (Fig. 6B). Conversely, nematodes exposed to PA14 Δ*amiE* strains died as quickly as those fed with PA14 WT (Fig. 6B). Taken together, our data clearly show that overproduction of AmiE resulted in virulence decrease of *P. aeruginosa* in these two surrogate infection models.

### Involvement of AmiE in virulence in an *in vivo* mouse infection model

**Figure 7:**
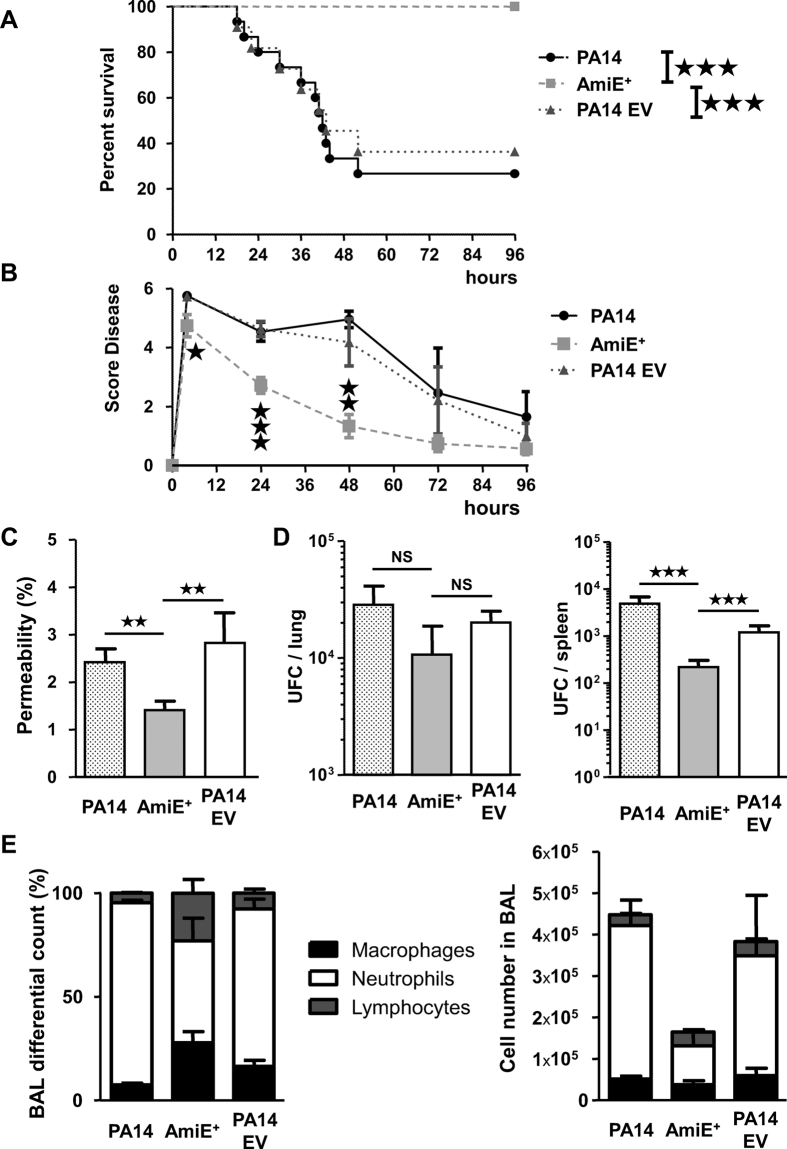
Effects of *P. aeruginosa* AmiE^+^ strain on acute lung injury model. (**A** and **B**) Mice were infected with intranasal instillation of 1.10^7^ CFU of *P. aeruginosa* PA14 WT, PA14 EV and AmiE^+^ strains (n = 11). (**A**) Lethality was monitored for 96 h after *P. aeruginosa* infection. (**B**) Score disease assessed the infection severity by evaluating mouse motility, mouse temperature, aspect of fur and weight loss. (**C, D** and **E**) Mice were infected with intranasal instillation of 5.10^6^ CFU of *P. aeruginosa* PA14 WT, PA14 EV and AmiE^+^ strains (n = 6). (**C**) Lung injury reflects the integrity of alveolar-capillary barrier measuring the leakage of FITC-labeled bovine serum albumin from the serum to the lung. (**D**) Bacterial load in the lungs and dissemination assessed through cultured spleen or cultured lung homogenate. (**E**) Cells from bronchoalveolar lavage (BAL) were counted to determine percentage distribution of immune stimulatory cells. ^★^*P* < 0.05; ^★★^*P* < 0.01; ^★★★^*P* < 0.001.

In agreement with our data on cells (Fig. 5C) as well as on amoeba (Fig. 6A) or nematodes (Fig. 6B), we observed that AmiE^+^ was totally non-virulent in a murine acute lung injury model. Indeed, at 96 h post infection all AmiE^+^ inoculated mice were still alive compared to only 36% in the case of PA14 EV treated-mice (Fig. 7A). The analysis of various parameters enabling to score the infection severity showed that the inoculation of either PA14 WT or PA14 EV induced rapidly (4 h) a phenotype of infection that subsequently slowly decreased until the end of the experiment (96 h) (Fig. 7B). AmiE^+^ infected mice initially presented a score disease slightly lower than the control strains (*P* < 0.05 versus PA14EV strain) after 4 h (Fig. 7B), but the score disease continuously fell after 4 h and was strongly reduced at 24 h (*P* < 0.001) and 48 h (*P* < 0.01) (Fig. 7B).

Lung injury was further assessed using the lung permeability index, mediated by sub-lethal inocula of PA14 WT, AmiE^+^ and PA14 EV strains (Fig. 7C). Mice infected with the AmiE^+^ strain showed lower lung permeability than the control strains (Fig. 7C), whereas the numbers of bacteria present in the lungs were not significantly different for the three strains (Fig. 7D). In contrast, the bacterial cell counts in the spleens were lower for the AmiE^+^ strain compared to both PA14 WT and PA14 EV strains (*P* < 0.001) (Fig. 7D). It is interesting to note that the growth of the AmiE+ strain is improved compared with both control strains, PA14 WT and PA14 EV, suggesting that AmiE over-production has no adverse effect on growth (Supplementary Figure S5). In parallel, we observed that the distribution of immune cells recruited into the lungs was different between mice infected with AmiE^+^ and mice infected with control strains (Fig. 7E). More precisely, neutrophils were in lower proportion in the lungs, whereas both macrophages and lymphocytes were in higher proportion in AmiE^+^ -infected mice compared to control strain- infected mice (Fig. 7E).

## Discussion

In previous studies, we showed that the opportunistic pathogen *P. aeruginosa* is able to sense the human C-type natriuretic peptide (CNP), which leads to modifying its biofilm formation^12^,^16^. This responsiveness of *P. aeruginosa* to this human hormone is relayed by the specific bacterial sensor protein AmiC, which displays a structural and pharmacological profile identical to the human natriuretic receptor subtype C (hNPR-C)^12^. We observed that the binding of CNP to AmiC enhances the transcription of the *ami* operon, including the *amiE* gene^12^ which encodes the AmiE amidase. The aim of the present study is to further understand if AmiE, so far known as an enzyme involved in *P. aeruginosa* secondary metabolism, could be involved in biofilm formation and bacterial virulence regulation. For this purpose we constructed an AmiE^+^ strain in which the AmiE enzyme is 11.8 more produced as compared with the wild type, mimicking what it happens in *P. aeruginosa* biofilm where the production of this enzyme is known to be strongly enhanced^29^,^30^.

Using dynamic conditions (i.e. flow cell system), we previously demonstrated that CNP strongly decreases *P. aeruginosa* biofilm formation^12^. The involvement of AmiC sensor protein in this effect was confirmed by the fact that a strain which was unable to produce AmiC (Δ*amiC*) not only lost its sensitivity to CNP, but also was strongly impaired in biofilm formation under dynamic conditions^12^.

The *P. aeruginosa ami* operon is composed of five genes in *P. aeruginosa* PAO1 (*amiEBCRS*) and of four genes in *P. aeruginosa* PA14 (*amiEBCR*) [ www.pseudomonas.com]^31^. AmiC activation by its known ligand acetamide^11^, or CNP^12^ is known to cause the release of AmiR leading to the transcriptional activation of the entire *ami* operon^14^,^32^. Therefore, in the *ΔamiC* mutant strain, we speculated that the AmiR regulator may be free and could induce the *ami* operon transcription, thereby enhancing the level of the AmiE protein. This suggested that over-expression of the *amiE* gene could mimic both CNP-induced and *ΔamiC* mutant phenotypes. In the present study, we validated this hypothesis by showing that over-production of AmiE reduces the ability of the bacteria to form a biofilm on glass in dynamic conditions and prevents the shaping of typical mushroom-like structures. To better understand the involvement of AmiE in biofilm formation, we investigated the motility of both *ΔamiE* and AmiE^+^ strains since biofilm formation is related to motility^33^,^34^. We showed here that AmiE is partially required for swarming in *P. aeruginosa*. In line with the increased swarming motility, we observed that the AmiE^+^ strain over-produced RhlA by about 6.5 fold compared to the PA14 EV strain. Interestingly, RhlA is a key enzyme that is required in synthesis of rhamnolipids, which are needed for swarming motility^35^. Likewise, we showed that an excess of the AmiE enzyme favours rhamnolipid production in a range comprised between 3 to 8-fold, contributing to the AmiE^+^ hyper-swarmer phenotype. By contrast, three major type 4 pilus proteins were under-produced in AmiE^+^ explaining its strongly affected twitching motility.

The hyper-swarming phenotype of AmiE^+^ suggested moreover a potential role of AmiE in virulence regulation. Since it is admitted that swarming and virulence are inversely regulated^36^, we evaluated the impact of AmiE over-production on cytotoxicity and virulence using several infection models. Whereas the absence of AmiE had no impact on virulence, an overproduction of AmiE severely affected *P. aeruginosa* virulence. The analysis of the virulence activity of AmiE^+^ strain in mouse revealed that, whereas the bacteria were able to colonize the lungs as efficiently as the wild type strain bacteria, the increased production of AmiE resulted in a decrease of neutrophils recruitment in the lungs. Since it was shown that neutrophils detected *P. aeruginosa* by sensing either the bacterial 3-oxo-C_12_-HSL molecule^37^ through a specific receptor^38^ or the PQS signalling molecule^39^, our data suggested that AmiE could be involved in the regulation of at least some if not all *P. aeruginosa* QS signaling compounds. In the present study, we observed that after a temporary enhancement of QS compound production through an increase of both LasR and RhlR amounts, high levels of AmiE caused a strong inhibition of 3-oxo-C_12_-HSL production after 24 h. This reduction of 3-oxo-C_12_-HSL production, a major factor in *P. aeruginosa* virulence regulation, could partly explain the observed loss of virulence activity of the AmiE^+^ strain. Interestingly, it has been recently described *in vitro* that *Acinetobacter* sp. strain Ooi24 expresses an *N*-acylhomoserine lactone acylase belonging to the amidase family (named also AmiE in this bacterium) that degrades specifically 3-oxo-C_12_-HSL compound^40^ suggesting that *P. aeruginosa* AmiE could directly degrade this QS signal. However, so far, no acylase activity could be assigned to *P. aeruginosa* AmiE. In addition, it is interesting to note that in *P. aeruginosa* AmiE over-production caused a major down-regulation by a factor 13 of the S-adenosylmethionine decarboxylase PA14_63110 (Supplementary Figure S1A), an enzyme that is involved in producing the precursor of the HSL moiety S-adenosylmethioninamine^41^. This could explain the strong production level decrease of 3-oxo-C_12_-HSL. In parallel it is interesting to note that retrieving AmiE’s *in silico* functional interactions from STRING database^42^, led to predict interactions of AmiE with PA14_63110, as well as with the guanidinobutyrase (GbuA), a mutant of which was shown to be strongly affected in PQS production^43^. Remarkably, AmiE over-production induced also a decrease in PQS synthesis at least through a lower accumulation of i) its biosynthetic enzymes PqsB, PqsC and PqsD, and ii) the kynunerinase KinU (Supplementary Table S1) that is involved in the degradation of kynurenine leading to the production of the PQS precursor anthranilate^44^. Taken together, these data suggest strong relationships between AmiE and QS signals production, thus linking metabolism and QS in *P. aeruginosa*, as previously suggested^43^.

Since the AmiE^+^ strain appeared to be much less aggressive in a mouse lung infection model, this prompted us to evaluate the ability of the strains to form microcolonies in liquid artificial sputum medium (ASM), which mimics the mucus from the lungs of cystic fibrosis affected patients^45^. In this condition, we observed that the AmiE^+^ strain was impaired in the formation of tight microcolonies as compared to the wild type, suggesting a possible alteration of the self-aggregation phenotype in ASM medium. This phenotype could be related to the strongly decreased production of type IV pili and of numerous proteins that are involved in LPS structure (Wzz, OrfM, OrfK, OrfH) (Fig. 1B and Supplementary Table S1), since these factors have been shown to be involved in tight micro-colony formation in ASM medium^45^. In addition, the increased production of rhamnolipids that we demonstrated would also result in increased dispersion of the AmiE^+^ cells in ASM medium.

The whole proteome analysis of the AmiE over-producing strain revealed a down-regulation of a couple of important bacterial virulence actors. Among the more relevant proteins down-regulated in AmiE^+^ and involved in *P. aeruginosa* virulence, there are several proteins involved in LPS synthesis (UDP-N-acetylglucosamine 2-epimerase, UDP-N-acetyl-D-mannosaminuronate dehydrogenase and O-antigen chain length regulator)^46^ (see Supplementary Table S1). It is interesting to note that the hormone CNP, which binds to AmiC^12^ is also able to modify *P. aeruginosa* LPS structure^47^. Other down-regulated proteins include proteins involved in pilus synthesis^46^, the alkaline metalloproteinase^46^, the hydrogen cyanide synthesis protein HcnC, proteins involved in the quinolone signal synthesis and one protein involved in type II secretion (T2SS)^48^ (Supplementary Table S1 and Supplementary Figure S1). In addition, it is interesting to note that we identified 31 proteins annotated as hypothetical proteins in PA14, among which 20 possess an orthologue characterized in PAO1 strain (http://www.pseudomonas.com)^31^, presenting an altered concentration in AmiE^+^ strain as compared with the control strain. Therefore, we cannot exclude that among these 31 proteins, one of them could be involved in virulence regulation, including for the 11 proteins without PAO1 orthologue counterpart.

Our previous study showing that the hormone CNP is able to bind AmiC triggering thus both *ami* operon transcription and an enhancement of the bacterial virulence^16^ could not directly fit with our present data showing that AmiE, when over-produced, negatively controls virulence in *P. aeruginosa*. Next to the direct control of *amiE* transcription by the AmiC/AmiR sensor/regulator pair, it has been shown that the *amiE* gene translation is under the Crc/Hfq proteins control^49^. More precisely, Crc and/or Hfq after binding to *amiE* RNA blocks *amiE* translation, down-regulating AmiE protein production^15^,^18^. Numerous studies have been performed using a Δ*crc* mutant strain to determine the targets of this protein. In this way, it has been shown that bacteria lacking Crc were affected in virulence towards *D. discoideum*^50^, and in the expression of virulence factors including ToxA, PlcB and protease IV^51^, in biofilm formation^52^,^53^, motility through type IV pilus biogenesis^52^, and in PQS synthesis^49^. All these phenotypes are modified in the same way in the AmiE^+^ strain used in the present study, suggesting that these phenotypes of Δ*crc* are at least in part due to an over-production of AmiE via an enhanced translation of the *amiE* mRNA reinforcing our hypothesis that AmiE could have additional functions than those previously identified.

We have previously shown that CNP slightly enhances the bacterial virulence^16^ through *ami* operon transcription^12^. Because CNP triggers AmiR release after binding to AmiC sensor, it is tempting to speculate that the transcription anti-termination factor AmiR could regulate other targets involved in virulence promotion and biofilm repression, conferring to the sensor AmiC protein, in association with its AmiR partner new functions. In this context, the competition between the Crc and/or Hfq proteins which bind RNA^18^ and the AmiR RNA-binding positive regulator should be considered.

Taken together our data suggest that the *P. aeruginosa* AmiE protein could exercise additional functions in relation with the bacterial virulence control and biofilm formation. The activation of AmiE after binding of CNP on AmiC^12^ in addition to the anti-biofilm activity of the CNP peptide could open up an opportunity for the development of new anti-*Pseudomonas aeruginosa* therapeutic approaches, particularly in the case of cystic fibrosis. This hypothesis is supported by the fact that a natriuretic peptide receptor agonist is already used as bronchodilator drug^54^. Since after activation of AmiC both bacterial virulence and biofilm formation are impacted, the understanding of the cellular pathways involving the AmiR regulator and AmiE could be very helpful in a therapeutic context.

## Methods

### Ethics Statement

All experimentations involving animals were carried out in compliance with French and European regulations on the care and protection of laboratory animals (European Commission Directive 86/609 and the French Act #2001–486, issued on June 6, 2001) and performed by certified personnel. The study and all experimental protocols associated were registered and approved by the French authorities (Ministère de l’Enseignement Supérieur et de la Recherche - Direction Générale pour la Recherche et l’Innovation - Secrétariat Autorisation de projet, registration number 00481.01). Animals were housed at the Lille University Animal Research Facility (Département Hospitalo-Universitaire de Recherche Expérimentale de Lille, France) accredited by the French Ministry of Agriculture for animal care and use in research (#B59–350009).

### Bacterial strains and bacterial cultures

All strains used in this study are listed in Supplementary Table S2. Briefly, *P. aeruginosa* PA14 wild type strain and PA14 Δ*amiE* mutant strain were obtained from Harvard Medical School (Boston, MA)^55^ and kindly provided by the Biomerit Research Center (Univ. Cork, Ireland). The *P. aeruginosa* PA14 AmiE^+^ strain, developed for this study by the LMSM, was obtained by transformation with the pBBR-MCS5 plasmid^56^ containing the *amiE* gene. We used the following primers for amplifying the *amiE* gene: (Forward primer) TAA TAA AAG CTT ACA AGA GGT GAT ATC CAT GCG T (including only the last 16 nt upstream of *amiE* coding sequence) and (Reverse primer) TAA TAA TCT AGA ATG TCG CTC AGA AAA GGC AT. The choice of the forward primer allowed to conserve the ribosome binding site while deleting about half of the Hfq binding sequence from the *amiE* mRNA^18^ to reduce Hfq repression of *amiE* translation. The two enzymes *Hin*dIII and *Xba*I were used for cloning the *amiE* amplicon into pBBR1-MCS5. All strains were grown at 37 °C in Luria Bertani medium (LB). We observed that PA14 wild type strain, PA14 strain harbouring the pBBR1-MCS5 alone (PA14 EV) and PA14 Δ*amiE* mutant strain grew exactly with the same kinetics. In contrast, the culture of the strain that overproduced AmiE (AmiE^+^) reached a maximum OD_580_ value that was 19% higher as compared to cultures of the three other strains (Supplementary Figure S5). For this reason data presented in this manuscript were divided by the OD measured.

### Whole proteome identification and quantification

Three independent cultures for both PA14 WT, PA14 EV and AmiE^+^ strains were realized using an inoculum of 0.08 (OD_580_). After 5 hours of growth, bacteria were centrifuged (7,000 g; 10 min; 4 °C) and the supernatants were removed. Bacterial pellets were re-suspended in IEF buffer (7 M urea, 2 M thiourea, 2 mM TBP; 20 mM DTT, 2% CHAPS and 0.5% NP40) and sonicated. Bacterial lysates were centrifuged (10,000 g; 10 min; 4 °C). Protein concentrations in the supernatants were estimated by Bradford protein assay and 25 μg of protein were loaded on a 7% SDS-PAGE stacking gel and a brief electrophoresis was run. The gel was stained with Coomassie blue, the staining was then eliminated and the protein band was recovered. The protein band was washed with water and successively soaked in 5 mM dithiothreitol and 10 mM iodoacetamide. The bands were dehydrated by successive bath in acetonitrile (ACN). Proteins were digested overnight by trypsin in 10 mM ammonium bicarbonate at pH 7.8 and 37 °C. Trypsin was inactivated with 0.1% of TFA. Peptides were extracted from gel bands by immersing in ACN, dried and stored at −20 °C until Orbitrap analysis.

All experiments were performed on a LTQ-Orbitrap Elite (Thermo Scientific) coupled to an Easy nLC II system (Thermo Scientific). One microliter of sample was injected onto an enrichment column (C18 PepMap100, Thermo Scientific). The separation was performed with an analytical column needle (NTCC-360/100-5-153, NikkyoTechnos, Japan). The mobile phase consisted of H_2_O/0.1% formic acid (FA) (buffer A) and CH_3_CN/FA 0.1% (buffer B). Tryptic peptides were eluted at a flow rate of 300 nL/min using a three-step linear gradient: from 2 to 40% B over 75 min, from 40 to 80% B in 4 min and 11 min at 80% B. The mass spectrometer was operated in positive ionization mode with capillary voltage and source temperature set at 1.5 kV and 275 °C, respectively. The samples were analyzed using CID (collision induced dissociation) method. The first scan (MS spectra) was recorded in the Orbitrap analyzer (R = 60,000) with the mass range m/z 400–1800. Then, the 20 most intense ions were selected for MS^2^ experiments. Singly charged species were excluded for MS^2^ experiments. Dynamic exclusion of already fragmented precursor ions was applied for 30 s, with a repeat count of 1, a repeat duration of 30 s and an exclusion mass width of ± 10 ppm. Fragmentation occurred in the linear ion trap analyzer with collision energy of 35%. All measurements in the Orbitrap analyzer were performed with on-the-fly internal recalibration (lock mass) at m/z 445.12002 (polydimethylcyclosiloxane).

After MS analysis, raw data were imported in Progenesis LC-MS software (Nonlinear Dynamics). For comparison, one sample was set as a reference and the retention times of all other samples within the experiment were aligned. After alignment and normalization, statistical analysis was performed for one-way analysis of variance (ANOVA) calculations. Peptide features presenting a p-value and a q-value less than 0.05, and a power greater than 0.8 were retained. MS/MS spectra from selected peptides were exported for peptide identification with Mascot (Matrix Science) against the database restricted to *P. aeruginosa* PA14 (http://www.pseudomonas.com). Database searches were performed with the following parameters: 1 missed trypsin cleavage site allowed; variable modifications: carbamidomethylation of cysteine and oxidation of methionine. Peptides with scores above 20 were imported into Progenesis. For each condition, the total cumulative abundance of the protein was calculated by summing the abundances of peptides. Proteins identified with less than 2 peptides were discarded. Only the proteins which varied by 2-fold in these average normalized abundances between growth conditions were retained.

### Biofilm formation under dynamic condition

Biofilms were grown at 37 °C in a three-channel flow cell with individual channel dimensions of 1 mm × 4 mm × 40 mm (Biocentrum, DTU, Danemark)^57^, using a microscope coverslip (ST1, VWR) as substratum. 1 ml of bacterial suspension at OD_600_ = 0.1 was injected into a flow cell channel, and bacteria were allowed to adhere to the glass coverslip for 2 h. LB medium was then pumped with a 1.5 ml h^−1^ flow at 37 °C during 24 h. At the end of the experiments, the biofilms were stained with 5 μM Syto 61 red dye (Molecular Probes). Observations were made using a confocal laser scanning microscope (LSM 710 confocal laser-scanning microscope; Zeiss) using a 40× oil immersion objective. The biofilm thicknesses (μm) and corresponding biovolumes (in μm^3^/μm^2^) were estimated by measuring field samples using COMSTAT software^58^.

### Rhamnolipids analysis by liquid chromatography-mass spectrometry (LC-MS)

Cultures of *P. aeruginosa* were grown in LB medium at 37 °C under shaking condition. After 24 h incubation, bacteria were removed by centrifugation at 10,000 g for 10 min. Supernatants were filtrated and stored at −20 °C until analysis.

Supernatants were analyzed using a Liquid chromatography–mass spectrometry Quadrupole Time-of-Flight (LC-MS-Q-TOF) (LC: Ultimate 3000, Dionex, MS: microTof Q, Bruker, Germany) system. Chromatography separation was achieved after injection of 20 μL of supernatant on a C18 column (200*2 mm, 5 μm). The column and sample temperatures were 30 °C and 15 °C, respectively. Flow rate was 0.2 mL/min with a total run of 43 min. The mobile phase consisted of water/acetonitrile 65/35 (A) and water/acetonitrile 10/90 (B), both containing 4 mM ammonium acetate. The program corresponded to an isocratic elution of 100% A for the initial 4 min, followed by a linear gradient 0–27% B for 5 min, an isocratic elution for 6 min and a linear gradient of 27–100% B for 15 min. Sample detection was performed in the negative mode by the Q-TOF mass spectrometer. This hybrid triple quadrupole time-of-flight was equipped with an electrospray source. Source conditions were the following: nebulizer 40 psi, dry gas 9 L/min, and temperature 200 °C. The scan range was 50–1000 *m*/*z*.

### ***N***-acyl-homoserine lactone (AHL) assays

*C. violaceum* CV026 was used as a biosensor to visualize relative AHL production by *P. aeruginosa* strains^23^. Seven milliliter volumes of LB agar (1.5%) were seeded with 100 μl of an overnight LB culture of *C. violaceum* CV026 and poured immediately over the surface of pre-warmed LB agar plates prepared in Petri dishes. When the overlaid agar had solidified *P. aeruginosa* strains were picked on the surface. Violacein halo production was observed after 24 h incubation at 37 °C.

Cultures of *P. aeruginosa* were grown in LB under shaking condition at 37 °C. At the end of the logarithmic phase (5 h) and during the stationary phase (24 h), 20 ml of cultures were harvested and centrifuged for 10 min, 10,000 g and 4 °C. The supernatants (20 ml) were extracted twice with equal volumes of ethyl acetate. The combined extracts were dried over anhydrous magnesium sulfate, evaporated to dryness, dissolved in 500 μl of high performance liquid chromatography (HPLC)-grade acetonitrile (Fisher Scientific, France) and stored at −20 °C until analysis.

The synthetic standards C_4_-HSL and 3-oxo-C_12_-HSL (Sigma-Aldrich, France), and stock solutions prepared in HPLC-grade ethyl acetate (Fisher Scientific, France) were stored at −20 °C. Concentrated extracts were analyzed by Liquid Chromatography-Ultra Violet Detection-High Resolution Mass Spectrometry LC-UV-HRMS. LC-UV-HRMS experiments were carried on a micrOTOF-Q II apparatus (BruckerDaltonics, Germany) operating in positive electrospray mode in full scan from *m/z* = 50 to *m/z* = 800 with a theoretical mass accuracy of 2 ppm coupled to an Agilent Technologies Series 1100 vacuum degasser, LC pump and autosampler (Hewlett-Packard, Germany) equipped with a C6-Phenyl column 250 mm × 4.6 mm × 5 μm (Phenimenex, Germany). Gradient was as follows: water/acetonitrile (95/5, v/v) added to 10 mM ammonium acetate was linearly increased to acetonitrile 100% added to 10 mM of ammonium acetate during 15 min, then held for 6 min. Flow was kept constant at 600 μL/min and column temperature settled at 40 °C. Identification was confirmed an the basis of the detection of the pseudomolecular ion [M + H]^+^ and/or sodium adduct [M + Na]^+^ and lactone ion [C_5_H_12_NO]^+^ (*m/z* = 102.0550) with *m/z* values varying from less than ±0.02 Da.

### Amoeba plaque assay

The amoeba plaque assay was performed as previously described^59^. Briefly, 50 μl of an overnight bacterial culture grown in LB medium was mixed with 200 μl PBS, plated on M9 agar plate and dried under a laminar flow bench for 1 h. *Dictyostelium discoideum* Ax2 grown for 2 to 4 days in HL-5 medium were harvested by centrifugation, washed and gently re-suspended in PBS. Amoebae were adjusted to a concentration of 8 × 10^6^ cells/ml and kept on ice before use. This stock solution was used to prepare droplets of 5 μl containing between 5 and 40,000 amoebae, which were subsequently spotted onto the bacterial lawn. The plates were incubated for 5 days at 22.5 °C and the highest dilution at which growth of amoebae caused a visible clear plaque due to bacterial clearance was considered as the test end point. Ten independent experiments were performed for each bacterial strain.

### *Caenorhabditis elegans* virulence assay

The *Caenorhabditis elegans* wild type Bristol strain N2 was obtained from the *Caenorhabditis* Genetics Center (Minneapolis, MN, USA). *C. elegans* were maintained under standard culturing conditions at 22 °C on nematode growth medium agar plates with *E. coli* OP50 as a food source^60^. Synchronous worm cultures were generated after exposure of an adult worm population to a sodium hypochlorite/sodium hydroxide solution and simultaneous egg hatching, as previously described^61^. Worms were incubated at 22 °C on an *E. coli* OP50 lawn until reaching the L4 (48 hours) life stage (confirmed by light microscopy).

Lawns used for *C. elegans* survival assays were prepared by spreading 50 μl of *P. aeruginosa* (control or mutant strains) on 35 mm conditioned Petri dishes supplemented with 0.05 mg/ml 5-fluoro-2′-deoxyuridine^16^. The plates were incubated overnight at 37 °C. Fifteen to twenty L4 synchronized worms were harvested with M9 solution (3 g KH_2_PO_4_, 6 g NaHPO_4_, 5 g NaCl, 1 ml 1 M MgSO_4_, H_2_O in 1 litre), placed on the 35 mm assay Petri dishes and incubated at 22 °C. Worm survival was scored at 1 hour, 24 hours and each subsequent day, using an Axiovert S100 optical microscope (Zeiss, Oberkochen, Germany) equipped with a Nikon digital Camera DXM 1200 F (Nikon Instruments, Melville, NY, USA). The worms were considered dead when they remained static without grinder movements for 20 s or did not respond to light flashes. Results are expressed as a percentage of living worms. The results are the average of three independent assays.

### Hydrogen cyanide assay

Hydrogen cyanide concentration in bacterial culture medium was determined by the polarographic technique described by Blier *et al*.^62^. Bacterial cultures were centrifuged for 10 minutes at 8,000 *g* and the supernatants (culture medium) were filtered through a 0.22 μm filter to remove all bacterial cells. Experiments were carried out using borate buffer (pH = 10.2) as supporting electrolyte. The solution was purged with N2 to remove dissolved oxygen and then for 20 s more between each addition of cyanide potassium (KCN) that was used as an internal standard. A scan of the electric potential was carried out in the negative direction from −0.1 V to −0.5 V with a sweep rate of 10 mV/s. The pulse amplitude was 0.05 V with a pulse duration of 0.04 s. The peak height of cyanide was measured at −230 mV in a differential pulse mode and the cyanide concentration was determined by the addition of 4 successive aliquots of 100 mg/L KCN standards.

### Motility assay

Each motility assay was carried on specific media. Swarming motilities were tested on a medium made with EIKEN nutriment 8 g/l, EIKEN Agar 5 g/l (EIKEN Chemicals, Tokyo) and glucose 5 g/l. Swimming motility assays were carried on LB with 0.3% agar. For these two mobility assays, a single colony of *P. aeruginosa* was picked with tips and inoculated onto the swarming plate simply by touching the medium surface. Twitching motilities were tested on LB plates containing 1% agar, and inoculated with a single colony picked with tips through the plate to its bottom. Plates were then incubated at 37 °C for 16 h or 18 h. The degree of motility was evaluated by measuring the average length of dendrites produced for swarming, the total diameter of the colony for swimming. After revelation using violet crystal 1% coloration on the bottom of Petri dishes after medium being removed, the degree of twitching motility was evaluated by measuring diameter of the colony.

### Biofilm formation in sputum-like medium

Artificial sputum media (ASM) is a culture medium that mimics the composition of the CF patient’s sputum, including amino-acids, mucin and free DNA. *Pseudomonas aeruginosa* growth in ASM mimics the growth during CF lung infection, leading to the formation of self-aggregating structures called microcolonies. ASM was prepared as described by Sriramulu *et al*.^45^. ASM (1 ml) was distributed in 24-wells culture plates and inoculated to an OD_600_ = 0.05 with an overnight culture of *P. aeruginosa*. In the case of *ami* mutants and for pBBR-MCS5 plasmid carrier strains, gentamicin (15 μg/ml) was added to the medium. Plates were then incubated for 72 h at 37 °C under 150 rpm agitation, and tight microcolonies formation attached to sputum components were observed visually on black fields without magnification. Tests were made on three independent replicates.

### Pyocyanin production assay

*P. aeruginosa* PA14 was grown at 37 °C under 180 rpm agitation in LB medium. After 24 h incubation, bacteria were removed by centrifugation at 8,000 *g* for 10 min, and pyocyanin was extracted from 2 ml supernatant using 2 ml of chloroform and re-extracted from the chloroform phase with 1 ml of 0.5 M HCl. The pyocyanin concentration was determined by measurement of OD_520_ in the extraction solution.

### Acute lung injury model

Age- and gender-matched mice in the C57BL/6 J background had free access to a standard laboratory chow diet in a half-day light cycle exposure and temperature-controlled Specified-Pathogen Free (SPF) environment as determined by the FELASA recommendations. Acute respiratory tract infection model was induced by intranasal instillation of *P. aeruginosa*. C57BL6/J mice were lightly anesthetised with inhaled Isoflurane (Forene Abbott, Queensborough, Kent, United Kingdom), after which 50 μl of the bacterial solution were administered intranasally (5.10^6^ CFU of each strain) the inoculum was increased to 1.10^7^ CFU of each strain only for the survival analysis over 96 h. All mice were euthanized at 24 h. For bacterial culture, mouse lungs were homogenised in sterile containers with sterile isotonic saline. Lung and spleen homogenates were sequentially diluted and cultured on bromocresol purple agar plates for 24 h to assess the bacterial load. Finally, to assess the alveolar capillary permeability, transvascular transport of albumin-FITC was used to study endothelial permeability in lungs of mice. For bronchoalveolar lavage (BAL), lungs from each experimental group were washed with a total of 1.5 ml of sterile phosphate-buffered saline (PBS). Recovered lavage fluid was pooled and centrifuged (300 g for 10 min), the cellular pellet was washed twice with PBS. Disease intensity was assessed by a composite clinical score taking into account mouse motility (0: normal, 1: altered, 2: motionless), temperature (0: normothermia, 2: hypothermia) and aspect of hair (0: normal, 1: mild bristly, 2: bristly).

### Statistical analysis

For the *C. elegans* killing assay, nematode survival was calculated by the Kaplan-Meier method, and survival differences were tested for significance using the log rank test (GraphPad Prism version 4.0; GraphPad Software, San Diego, California, USA). For other results, the *t* test was used to compare the means within the same set of experiments, using Past 3.x software. For the *in vivo* experiments using mice, statistical analysis was carried out using Prism 6 software (GraphPad). One-way analysis of variance (ANOVA) followed by t-test was used for all comparisons.

## Additional Information

**How to cite this article**: Clamens, T. *et al*. The aliphatic amidase AmiE is involved in regulation of *Pseudomonas aeruginosa* virulence. *Sci. Rep.* **7**, 41178; doi: 10.1038/srep41178 (2017).

**Publisher's note:** Springer Nature remains neutral with regard to jurisdictional claims in published maps and institutional affiliations.

## Supplementary Material

Supplemental Table S1

Supplemental Table S2 and Figures

## References

[b1] Costerton J. W., Stewart P. S. & Greenberg E. P. Bacterial biofilms: a common cause of persistent infections. Science 284, 1318–22 (1999). 10334980 10.1126/science.284.5418.1318

[b2] Lyczak J. B., Cannon C. L. & Pier G. B. Lung infections associated with cystic fibrosis. Clin Microbiol Rev 15, 194–222 (2002). 11932230 10.1128/CMR.15.2.194-222.2002 PMC118069

[b3] Camilli A. & Bassler B. L. Bacterial small-molecule signaling pathways. Science 311, 1113–6 (2006). 16497924 10.1126/science.1121357 PMC2776824

[b4] Seal J. B., Alverdy J. C., Zaborina O. & An G. Agent-based dynamic knowledge representation of *Pseudomonas aeruginosa* virulence activation in the stressed gut: Towards characterizing host-pathogen interactions in gut-derived sepsis. Theor Biol Med Model 8, 33 (2011). 21929759 10.1186/1742-4682-8-33 PMC3184268

[b5] Galperin M. Y. A census of membrane-bound and intracellular signal transduction proteins in bacteria: bacterial IQ, extroverts and introverts. BMC Microbiol 5, 35 (2005). 15955239 10.1186/1471-2180-5-35 PMC1183210

[b6] Coggan K. A. & Wolfgang M. C. Global regulatory pathways and cross-talk control *Pseudomonas aeruginosa* environmental lifestyle and virulence phenotype. Curr Issues Mol Biol 14, 47–70 (2012). 22354680 PMC12747716

[b7] Gooderham W. J. & Hancock R. E. Regulation of virulence and antibiotic resistance by two-component regulatory systems in *Pseudomonas aeruginosa*. FEMS Microbiol Rev 33, 279–94 (2009). 19243444 10.1111/j.1574-6976.2008.00135.x

[b8] Rodrigue A., Quentin Y., Lazdunski A., Mejean V. & Foglino M. Two-component systems in *Pseudomonas aeruginosa*: why so many? Trends Microbiol 8, 498–504 (2000). 11121759 10.1016/s0966-842x(00)01833-3

[b9] Lesouhaitier O. . Gram-negative bacterial sensors for eukaryotic signal molecules. Sensors 9, 6967–90 (2009). 22399982 10.3390/s90906967 PMC3290508

[b10] Kunert A. . Immune evasion of the human pathogen *Pseudomonas aeruginosa*: elongation factor Tuf is a factor H and plasminogen binding protein. J Immunol 179, 2979–88 (2007). 17709513 10.4049/jimmunol.179.5.2979

[b11] Wilson S. A., Wachira S. J., Drew R. E., Jones D. & Pearl L. H. Antitermination of amidase expression in *Pseudomonas aeruginosa* is controlled by a novel cytoplasmic amide-binding protein. EMBO J 12, 3637–42 (1993). 8253087 10.1002/j.1460-2075.1993.tb06037.x PMC413639

[b12] Rosay T. . *Pseudomonas aeruginosa* Expresses a Functional Human Natriuretic Peptide Receptor Ortholog: Involvement in Biofilm Formation. MBio 6, e01033–15 (2015). 26307165 10.1128/mBio.01033-15 PMC4550695

[b13] Wilson S. A. & Drew R. E. Transcriptional analysis of the amidase operon from *Pseudomonas aeruginosa*. J Bacteriol 177, 3052–7 (1995). 7539417 10.1128/jb.177.11.3052-3057.1995 PMC176992

[b14] O’Hara B. P. . Crystal structure and induction mechanism of AmiC-AmiR: a ligand-regulated transcription antitermination complex. EMBO J 18, 5175–86 (1999). 10508151 10.1093/emboj/18.19.5175 PMC1171588

[b15] Sonnleitner E., Abdou L. & Haas D. Small RNA as global regulator of carbon catabolite repression in *Pseudomonas aeruginosa*. Proc Natl Acad Sci USA 106, 21866–71 (2009). 20080802 10.1073/pnas.pnas.0910308106 PMC2799872

[b16] Blier A. S. . C-type natriuretic peptide modulates quorum sensing molecule and toxin production in *Pseudomonas aeruginosa*. Microbiology 157, 1929–44 (2011). 21511763 10.1099/mic.0.046755-0 PMC3755537

[b17] Collier D. N., Spence C., Cox M. J. & Phibbs P. V. Isolation and phenotypic characterization of *Pseudomonas aeruginosa* pseudorevertants containing suppressors of the catabolite repression control-defective crc-10 allele. FEMS Microbiol. Lett. 196, 87–92 (2001). 11267761 10.1111/j.1574-6968.2001.tb10546.x

[b18] Sonnleitner E. & Blasi U. Regulation of Hfq by the RNA CrcZ in *Pseudomonas aeruginosa* carbon catabolite repression. PLoS Genet 10, e1004440 (2014). 24945892 10.1371/journal.pgen.1004440 PMC4063720

[b19] de la Fuente-Nunez C. . Inhibition of bacterial biofilm formation and swarming motility by a small synthetic cationic peptide. Antimicrob Agents Chemother 56, 2696–704 (2012). 22354291 10.1128/AAC.00064-12 PMC3346644

[b20] Shrout J. D. . The impact of quorum sensing and swarming motility on *Pseudomonas aeruginosa* biofilm formation is nutritionally conditional. Mol Microbiol 62, 1264–77 (2006). 17059568 10.1111/j.1365-2958.2006.05421.x

[b21] Kohler T., Curty L. K., Barja F., van Delden C. & Pechere J. C. Swarming of *Pseudomonas aeruginosa* is dependent on cell-to-cell signaling and requires flagella and pili. J Bacteriol 182, 5990–6 (2000). 11029417 10.1128/jb.182.21.5990-5996.2000 PMC94731

[b22] Tremblay J., Richardson A. P., Lepine F. & Deziel E. Self-produced extracellular stimuli modulate the *Pseudomonas aeruginosa* swarming motility behaviour. Environ. Microbiol 9, 2622–30 (2007). 17803784 10.1111/j.1462-2920.2007.01396.x

[b23] McClean K. H. . Quorum sensing and *Chromobacterium violaceum*: exploitation of violacein production and inhibition for the detection of N-acylhomoserine lactones. Microbiology 143, 3703–11 (1997). 9421896 10.1099/00221287-143-12-3703

[b24] Morin D., Grasland B., Vallée-Réhel K., Dufau C. & Haras D. On-line high-performance liquid chromatography–mass spectrometric detection and quantification of N-acylhomoserine lactones, quorum sensing signal molecules, in the presence of biological matrices. J. Chromatogr. A 1002, 79–92 (2003). 12885081 10.1016/s0021-9673(03)00730-1

[b25] Ochsner U. A. & Reiser J. Autoinducer-mediated regulation of rhamnolipid biosurfactant synthesis in *Pseudomonas aeruginosa*. Proc Natl Acad Sci USA 92, 6424–6428 (1995). 7604006 10.1073/pnas.92.14.6424 PMC41530

[b26] Le Berre R. . Quorum-sensing activity and related virulence factor expression in clinically pathogenic isolates of *Pseudomonas aeruginosa*. Clin Microbiol Infect 14, 337–43 (2008). 18190582 10.1111/j.1469-0691.2007.01925.x

[b27] Dubern J. F. & Diggle S. P. Quorum sensing by 2-alkyl-4-quinolones in *Pseudomonas aeruginosa* and other bacterial species. Mol Biosyst 4, 882–8 (2008). 18704225 10.1039/b803796p

[b28] Mahajan-Miklos S., Tan M. W., Rahme L. G. & Ausubel F. M. Molecular mechanisms of bacterial virulence elucidated using a *Pseudomonas aeruginosa*-*Caenorhabditis elegans* pathogenesis model. Cell 96, 47–56 (1999). 9989496 10.1016/s0092-8674(00)80958-7

[b29] Park A. J. . A temporal examination of the planktonic and biofilm proteome of whole cell *Pseudomonas aeruginosa* PAO1 using quantitative mass spectrometry. Mol. Cell. Proteomics MCP 13, 1095–1105 (2014). 24532839 10.1074/mcp.M113.033985 PMC3977187

[b30] Park A. J. . Tracking the Dynamic Relationship between Cellular Systems and Extracellular Subproteomes in *Pseudomonas aeruginosa* Biofilms. J. Proteome Res. 14, 4524–4537 (2015). 26378716 10.1021/acs.jproteome.5b00262

[b31] Winsor G. L. . Enhanced annotations and features for comparing thousands of *Pseudomonas* genomes in the Pseudomonas genome database. Nucleic Acids Res 44, D646–53 (2016). 26578582 10.1093/nar/gkv1227 PMC4702867

[b32] Wilson S. & Drew R. Cloning and DNA sequence of amiC, a new gene regulating expression of the *Pseudomonas aeruginosa* aliphatic amidase, and purification of the amiC product. J Bacteriol 173, 4914–21 (1991). 1907262 10.1128/jb.173.16.4914-4921.1991 PMC208179

[b33] Klausen M., Aaes-Jorgensen A., Molin S. & Tolker-Nielsen T. Involvement of bacterial migration in the development of complex multicellular structures in *Pseudomonas aeruginosa* biofilms. Mol Microbiol 50, 61–8 (2003). 14507363 10.1046/j.1365-2958.2003.03677.x

[b34] O’Toole G. A. & Kolter R. Flagellar and twitching motility are necessary for *Pseudomonas aeruginosa* biofilm development. Mol Microbiol 30, 295–304 (1998). 9791175 10.1046/j.1365-2958.1998.01062.x

[b35] Deziel E., Lepine F., Milot S. & Villemur R. rhlA is required for the production of a novel biosurfactant promoting swarming motility in *Pseudomonas aeruginosa*: 3-(3-hydroxyalkanoyloxy)alkanoic acids (HAAs), the precursors of rhamnolipids. Microbiology 149, 2005–13 (2003). 12904540 10.1099/mic.0.26154-0

[b36] Tremblay J. & Deziel E. Gene expression in *Pseudomonas aeruginosa* swarming motility. BMC Genomics 11, 587 (2010). 20961425 10.1186/1471-2164-11-587 PMC3091734

[b37] Zimmermann S. . Induction of neutrophil chemotaxis by the quorum-sensing molecule N-(3-oxododecanoyl)-L-homoserine lactone. Infect Immun 74, 5687–92 (2006). 16988244 10.1128/IAI.01940-05 PMC1594900

[b38] Karlsson T., Musse F., Magnusson K. E. & Vikstrom E. N-Acylhomoserine lactones are potent neutrophil chemoattractants that act via calcium mobilization and actin remodeling. J Leukoc Biol 91, 15–26 (2012). 21807742 10.1189/jlb.0111034

[b39] Hansch G. M., Prior B., Brenner-Weiss G., Obst U. & Overhage J. The *Pseudomonas* quinolone signal (PQS) stimulates chemotaxis of polymorphonuclear neutrophils. J Appl Biomater Funct Mater 12, 21–6 (2014). 24829042 10.5301/jabfm.5000204

[b40] Ochiai S., Yasumoto S., Morohoshi T. & Ikeda T. AmiE, a novel N-acylhomoserine lactone acylase belonging to the amidase family, from the activated-sludge isolate *Acinetobacter* sp. strain Ooi24. *Appl Environ*. Microbiol 80, 6919–25 (2014). 10.1128/AEM.02190-14 PMC4249007 25172868

[b41] More M. I. . Enzymatic synthesis of a quorum-sensing autoinducer through use of defined substrates. Science 272, 1655–8 (1996). 8658141 10.1126/science.272.5268.1655

[b42] Szklarczyk D. . STRING v10: protein-protein interaction networks, integrated over the tree of life. Nucleic Acids Res 43, D447–52 (2015). 25352553 10.1093/nar/gku1003 PMC4383874

[b43] Jagmann N., Bleicher V., Busche T., Kalinowski J. & Philipp B. The guanidinobutyrase GbuA is essential for the alkylquinolone-regulated pyocyanin production during parasitic growth of *Pseudomonas aeruginosa* in co-culture with *Aeromonas hydrophila*. Environ. Microbiol 18, 3550–64, doi: 10.1111/1462-2920.13419 (2016). 27322205

[b44] Kasper S. H., Bonocora R. P., Wade J. T., Musah R. A. & Cady N. C. Chemical Inhibition of Kynureninase Reduces *Pseudomonas aeruginosa* Quorum Sensing and Virulence Factor Expression. ACS Chem Biol 11, 1106–17 (2016). 26785289 10.1021/acschembio.5b01082

[b45] Sriramulu D. D., Lunsdorf H., Lam J. S. & Romling U. Microcolony formation: a novel biofilm model of *Pseudomonas aeruginosa* for the cystic fibrosis lung. J Med Microbiol 54, 667–76 (2005). 15947432 10.1099/jmm.0.45969-0

[b46] Yang J., Chen L., Sun L., Yu J. & Jin Q. VFDB 2008 release: an enhanced web-based resource for comparative pathogenomics. Nucleic Acids Res 36, D539–42 (2008). 17984080 10.1093/nar/gkm951 PMC2238871

[b47] Veron W. . Natriuretic peptides affect *Pseudomonas aeruginosa* and specifically modify lipopolysaccharide biosynthesis. FEBS J 274, 5852–64 (2007). 17944935 10.1111/j.1742-4658.2007.06109.x

[b48] Filloux A. Protein Secretion Systems in *Pseudomonas aeruginosa*: An Essay on Diversity, Evolution, and Function. Front Microbiol 2, 155 (2011). 21811488 10.3389/fmicb.2011.00155 PMC3140646

[b49] Sonnleitner E. . Novel targets of the CbrAB/Crc carbon catabolite control system revealed by transcript abundance in *Pseudomonas aeruginosa*. PLoS One 7, e44637 (2012). 23115619 10.1371/journal.pone.0044637 PMC3480352

[b50] Linares J. F. . The global regulator Crc modulates metabolism, susceptibility to antibiotics and virulence in *Pseudomonas aeruginosa*. Environ. Microbiol 12, 3196–212 (2010). 20626455 10.1111/j.1462-2920.2010.02292.x

[b51] Reales-Calderon J. A., Corona F., Monteoliva L., Gil C. & Martinez J. L. Quantitative proteomics unravels that the post-transcriptional regulator Crc modulates the generation of vesicles and secreted virulence determinants of *Pseudomonas aeruginosa*. J Proteomics 127, 352–64 (2015). 26102536 10.1016/j.jprot.2015.06.009

[b52] O’Toole G. A., Gibbs K. A., Hager P. W., Phibbs P. V. & Kolter R. The global carbon metabolism regulator Crc is a component of a signal transduction pathway required for biofilm development by *Pseudomonas aeruginosa*. J Bacteriol 182, 425–31 (2000). 10629189 10.1128/jb.182.2.425-431.2000 PMC94292

[b53] Zhang L. . The catabolite repression control protein Crc plays a role in the development of antimicrobial-tolerant subpopulations in *Pseudomonas aeruginosa* biofilms. Microbiology 158, 3014–9 (2012). 23023972 10.1099/mic.0.061192-0

[b54] Edelson J. D. . *In vitro* and *in vivo* pharmacological profile of PL-3994, a novel cyclic peptide (Hept-cyclo(Cys-His-Phe-d-Ala-Gly-Arg-d-Nle-Asp-Arg-Ile-Ser-Cys)-Tyr-[Arg mimetic]-NH(2)) natriuretic peptide receptor-A agonist that is resistant to neutral endopeptidase and acts as a bronchodilator. Pulm Pharmacol Ther 26, 229–38 (2013). 23154072 10.1016/j.pupt.2012.11.001 PMC4070431

[b55] Liberati N. T. . An ordered, nonredundant library of *Pseudomonas aeruginosa* strain PA14 transposon insertion mutants. Proc Natl Acad Sci USA 103, 2833–8 (2006). 16477005 10.1073/pnas.0511100103 PMC1413827

[b56] Kovach M. E. . Four new derivatives of the broad-host-range cloning vector pBBR1MCS, carrying different antibiotic-resistance cassettes. Gene 166, 175–6 (1995). 8529885 10.1016/0378-1119(95)00584-1

[b57] Pamp S. J., Sternberg C. & Tolker-Nielsen T. Insight into the microbial multicellular lifestyle via flow-cell technology and confocal microscopy. Cytometry A 75, 90–103 (2009). 19051241 10.1002/cyto.a.20685

[b58] Heydorn A. . Experimental reproducibility in flow-chamber biofilms. Microbiology 146, 2409–15 (2000). 11021917 10.1099/00221287-146-10-2409

[b59] Breidenstein E. B. . The Lon protease is essential for full virulence in *Pseudomonas aeruginosa*. PLoS One 7, e49123 (2012). 23145092 10.1371/journal.pone.0049123 PMC3492299

[b60] Sulston J. & Hodgkin J. The Nematode *Caenorhabditis elegans*. (Wood, W. B., 1988).

[b61] Stiernagle T. Maintenance of *C. elegans. C. elegans* : a practical approach. WormBook 51–67, doi: 10.1895/wormbook.1.101.1 (1999).

[b62] Blier A. S. . Quantification of *Pseudomonas aeruginosa* hydrogen cyanide production by a polarographic approach. J Microbiol Methods 90, 20–4 (2012). 22537820 10.1016/j.mimet.2012.04.005

