# Supplementary material for: The aliphatic amidase AmiE is involved in regulation of Pseudomonas aeruginosa virulence
Source: Sci Rep. 2017 Jan 24;7:41178. doi: 10.1038/srep41178 (PMC5259723; doi:10.1038/srep41178)
Supplement: Supplemental Table S2 and Figures [file srep41178-s2.pdf]

Supplemental material figures and tables.

"The aliphatic amidase AmiE is involved in regulation of *Pseudomonas aeruginosa* virulence"

Thomas Clamens, Thibaut Rosay, Alexandre Crepin, Teddy Grandjean, Takfarinas Kentache, Julie Hardouin, Perrine Bortolotti, Anke Neidig, Marlies Mooij, Mélanie Hillion, Julien Vieillard, Pascal Cosette, Joerg Overhage, Fergal O'Gara, Emeline Bouffartigues, Alain Dufour, Sylvie Chevalier, Benoit Guery, Pierre Cornelis, Marc G.J. Feuilloley and Olivier Lesouhaitier

Table S2. Strains and plasmids used in this study.

Strain/Plasmid	Characteristics/modified gene	Reference or source
PA14	Wild type	Rahme *et al*., 1995
PA14 AmiE^{+}	PA14 over-expressing *amiE*	This study
PA14 EV	PA14 harboring empty vector	Rosay *et al* [12]
PA14 Δ*amiE*	*ΔamiE, ID 56575*	Liberati *et al*. [55]
pBBR-MCS5	Cloning Vector	Kovach *et al.* [56]
pBBR-MCS5::*amiE*	pBBR-MCS5 harboring *amiE*	This Study

Rahme, L.G. *et al*. Common virulence factors for bacterial pathogenicity in plants and animals. *Science* **268**, 1899–1902 (1995).

Supplemental Figure S1.

Relative protein amounts in AmiE+ strain (grey histogram) compared to the relative protein amounts in the control strain PA14 EV (white histograms), after 5 h of growth. Quantifications have been obtained from three independent experiments.

✶✶✶ = $P < 0.001$; ✶✶ = $P < 0.01$; NS = Not significantly different.

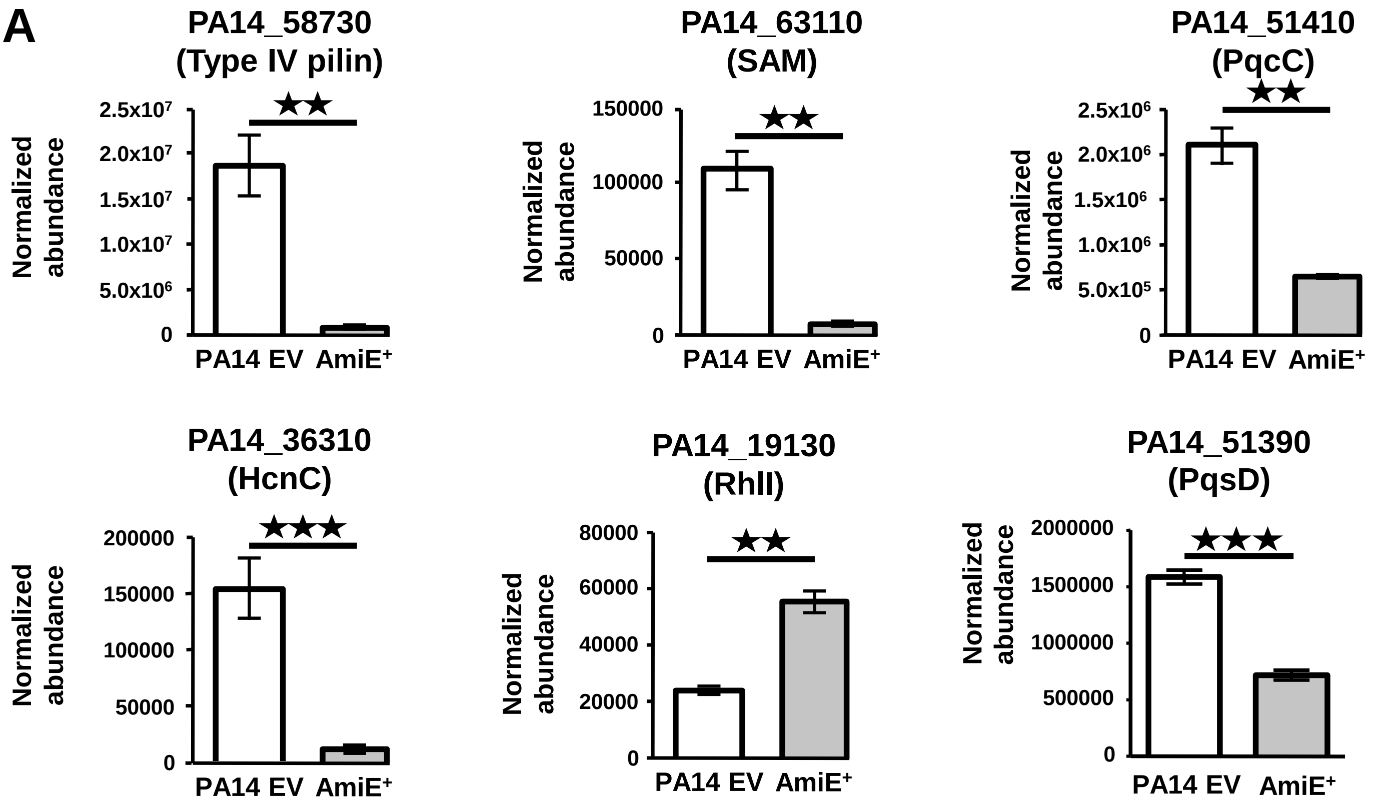

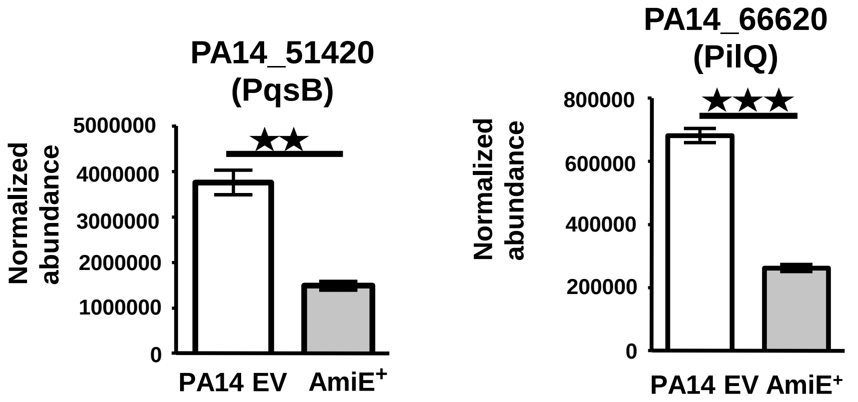

B

The total cumulative abundance of the protein RhlA was calculated by summing the abundances of only one peptide.

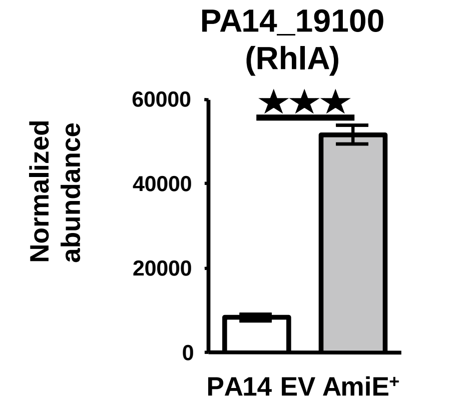

C Proteins showing a diffential expression level but not significantly different using the one-way analysis of variance (ANOVA).

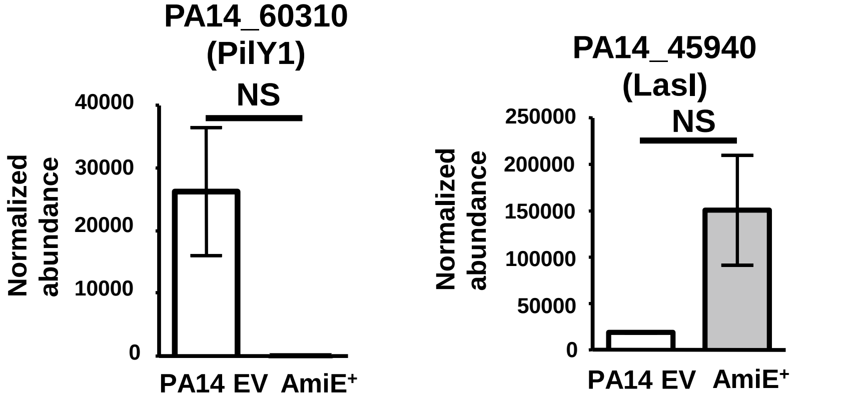

Supplemental Figure S2.

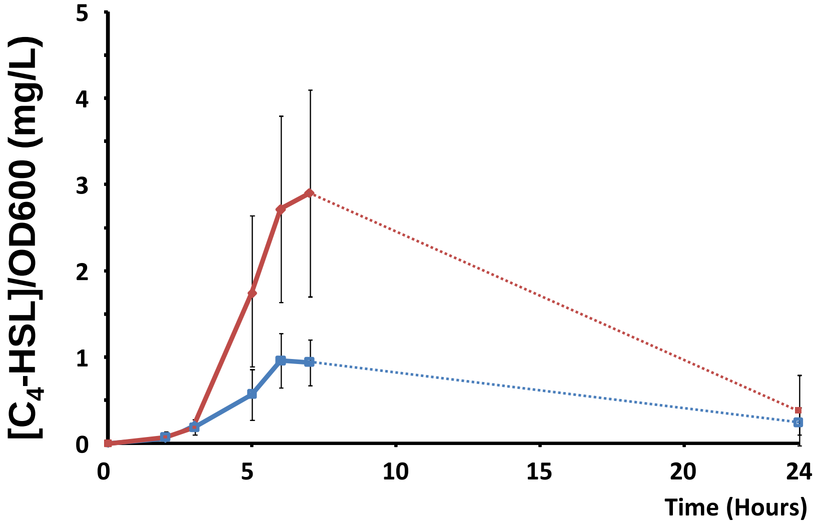

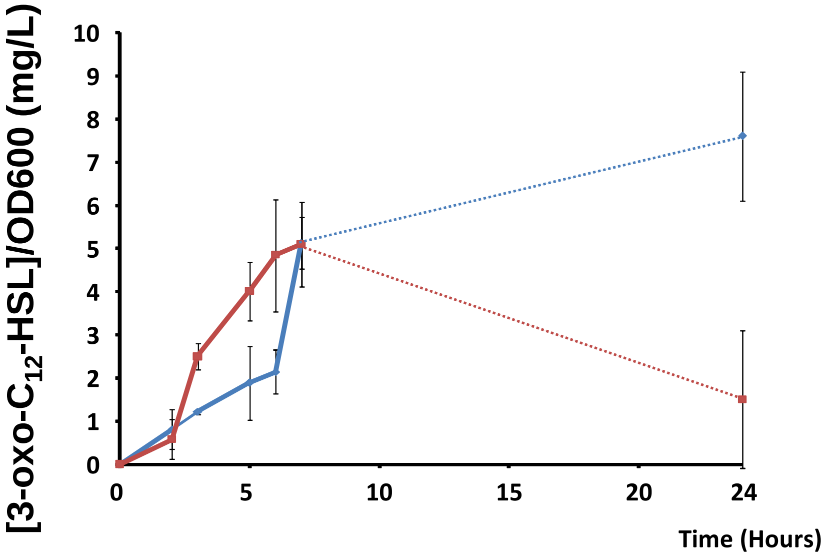

Supplemental Figure S3.

AmiE$^+$

PA14 EV

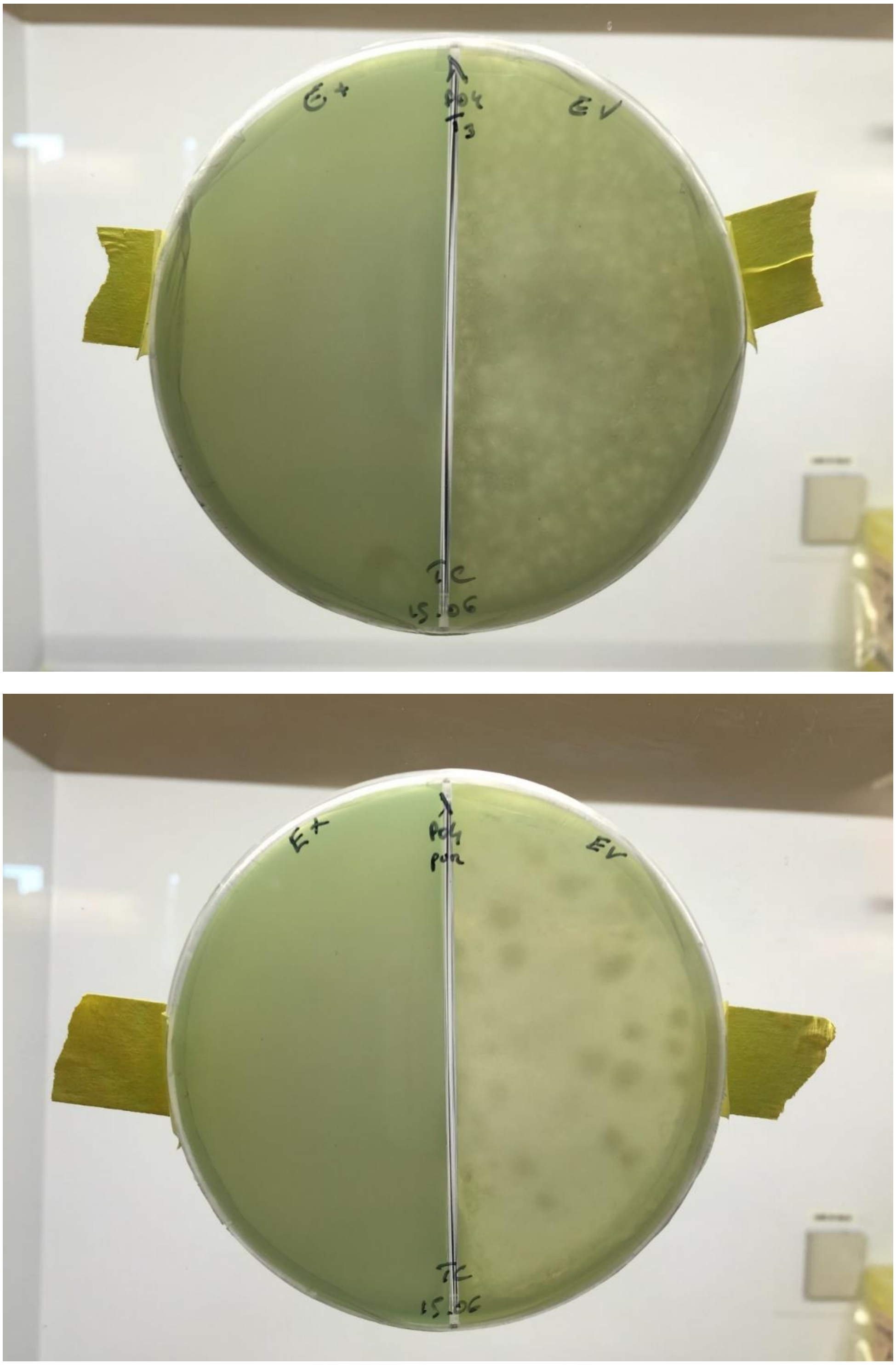

AmiE$^+$

PA14 EV

PO4 bacteriophage sensitivity assay

10^7 bacteria were mixed with ten-fold serial dilutions of bacteriophages ranging from 10^8 to 10^2 PFU (plaque forming unit) in 1.5mL of 0.5% Noble Agar and poured on top of 1.5% Agar Luria Bertani medium in a two-compartment Petri dish.

After 24h at 37°C, bacterial sensitivity to PO4 bacteriophages was estimated by counting the number of plaques.

Supplemental Figure S4.

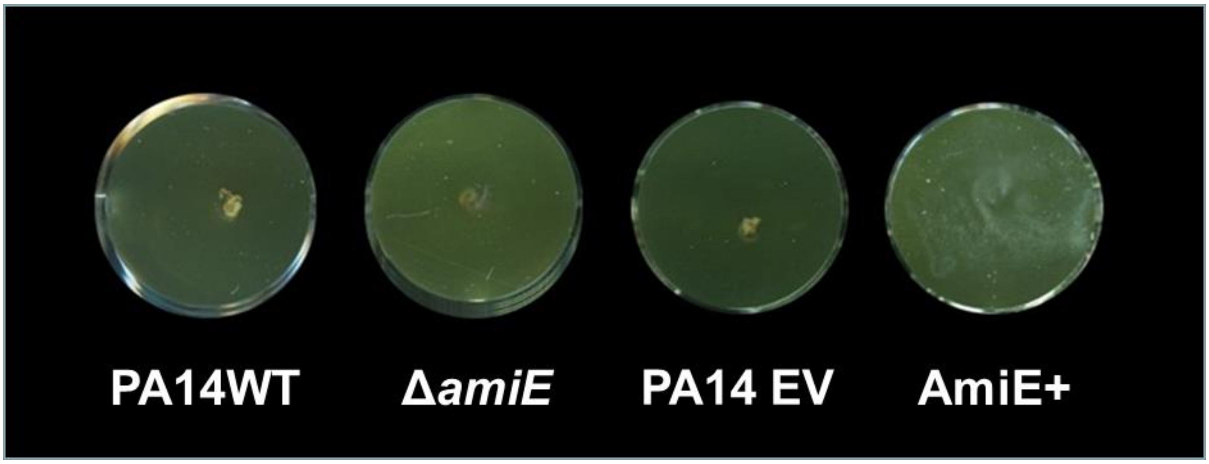

Microcolonies formation of *P. aeruginosa* PA14 strains in Artificial Sputum Medium (ASM). Tight microcolonies are visible in light beige at the bottom of the well. The images presented are representative of four independent experiments.

In artificial sputum-like medium, PA14 Δ*amiE* was able to produce similar microcolonies as PA14 WT, whereas PA14 $AmiE^{+}$ produced a more diffuse biofilm as compared with the PA14 EV strain.

Supplemental Figure S5.

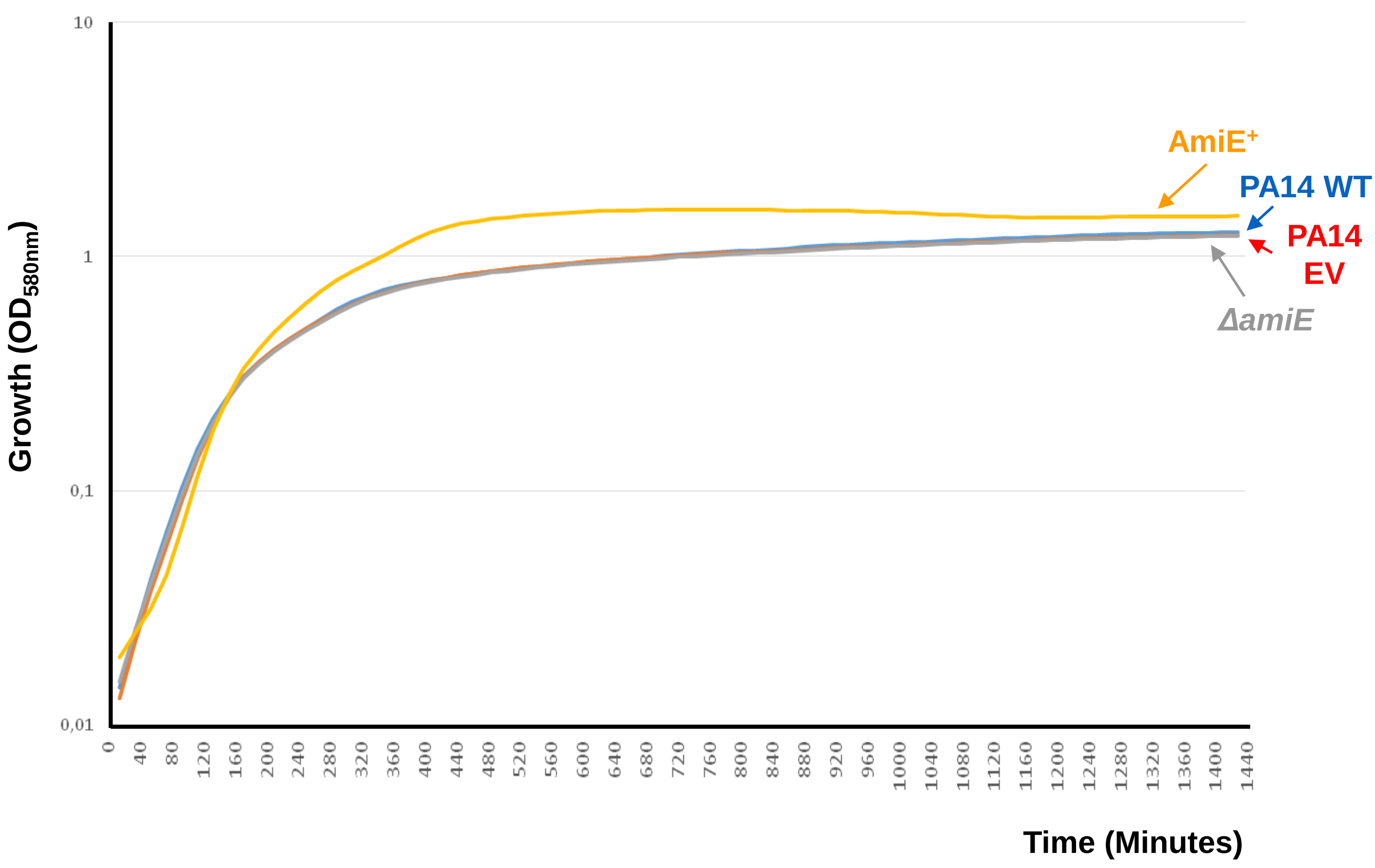

Culture growth curves (OD580nm)

Absorbance was recorded every 20 min for 24 hr. Curves are color-coded as follow: PA14 AmiE+ strain (orange), PA14 WT (blue), PA14 EV (red) and *ΔamiE* (gray). Results are the mean of twelve replicates from two independent experiments.
